# Syntheses and Study of a Pyrroline Nitroxide Condensed Phospholene Oxide and a Pyrroline Nitroxide Attached Diphenylphosphine

**DOI:** 10.3390/molecules26144366

**Published:** 2021-07-19

**Authors:** Mostafa Isbera, Balázs Bognár, Ferenc Gallyas, Attila Bényei, József Jekő, Tamás Kálai

**Affiliations:** 1 Faculty of Pharmacy, Institute of Organic and Medicinal Chemistry, University of Pécs, Szigeti st. 12, H-7624 Pécs, Hungary; mostafaisbera@gmail.com (M.I.); balazs.bognar@aok.pte.hu (B.B.); 2 Department of Biochemistry and Medical Chemistry, University of Pécs Medical School, H-7624 Pécs, Hungary; ferenc.gallyas@aok.pte.hu; 3 HAS-UP Nuclear-Mitochondrial Interactions Research Group, H-1245 Budapest, Hungary; 4 János Szentágothai Research Center, University of Pécs, Ifjúság 20, H-7624 Pécs, Hungary; 5 Department of Pharmaceutical Chemistry, University of Debrecen, Egyetem tér 1, H-4032 Debrecen, Hungary; benyei.attila@science.unideb.hu; 6 Department of Chemistry, University of Nyíregyháza, Sóstói st. 31/B, H-4440 Nyíregyháza, Hungary; jjozsi@gmail.com

**Keywords:** lithiation, McCormac reaction, nitroxides, protecting groups, reduction

## Abstract

The reaction of a diene nitroxide precursor with dichlorophenylphosphine in a McCormac procedure afforded 1,1,3,3-tetramethyl-5-phenyl-1,2,3,4,5,6-hexahydrophospholo[3,4-c]pyrrole-5-oxide-2-oxyl. Lithiation of the protected 3-iodo-pyrroline nitroxide followed by treatment with chlorodiphenylphosphine after deprotection afforded (1-oxyl-2,2,5,5-tetramethyl-2,5-dihydro-1*H*-pyrrol-3-yl)diphenylphosphine oxide, and after reduction, (1-oxyl-2,2,5,5-tetramethyl-2,5-dihydro-1*H*-pyrrol-3-yl)diphenylphosphine was realized, which was also supported by X-ray single crystal diffraction measurements. This pyrroline diphenylphosphine derivative was converted to hexadecylphosphonium salt, which is an analogue of antineoplastic agent, MITO-CP.

## 1. Introduction

Heterocyclic nitroxide derivatives of piperidine pyrrolidine, pyrroline and imidazoline have found various applications [1,2]. Stable nitroxide (aminoxyl) free radicals possess unique properties and reactivity, and despite decades of experience working with them, accessing new scaffolds without affecting the free valence is a challenge for every synthetic chemist active in this field [3]. Sometimes nitroxide moieties require temporary protection [4,5] to avoid irreversible destruction during various chemical transformations. Nitroxides have a wide range of applications; they are used as co-oxidants in organic chemistry, building blocks for magnetic materials, superoxide dismutase mimics, antiproliferative compounds, mediators of polymerization, redox active materials in batteries, magnetic resonance imaging (MRI), and electron paramagnetic resonance imaging (EPRI) contrast agents [1,2]. These various applications require numerous paramagnetic scaffolds adjusted to different utilizations. Our idea is to combine various phosphorus-containing functional groups with nitroxides, which may offer fascinating possibilities for synthetic, structural and biological studies and the utilization of these new compounds [6,7]. In continuation of our interest in the synthesis of nitroxide-based phosphorus compounds, we have promoted the synthesis of phosphorus-containing heterocycles condensed with pyrroline nitroxide and pyrroline nitroxide-diphenylphosphine and pyrroline nitroxide-diphenylphosphine oxide compounds. Although similar nitroxides have been previously reported with nitronyl nitroxides [8] and a phosphorus-containing heterocycle [9], our approach described in the present work might open a new route for synthesizing such novel types of paramagnetic phosphorus-containing compounds.

## 2. Results and Discussions

### 2.1. Synthesis of Paramagnetic Phospholene Oxide

**Scheme 1:**
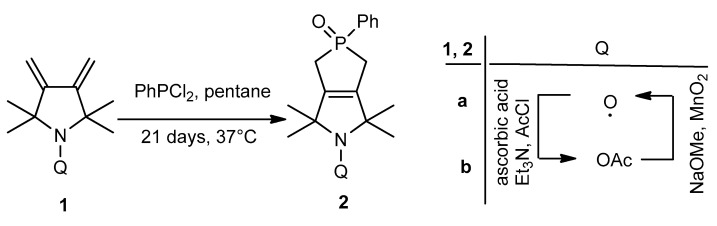
Synthesis of a paramagnetic phospholene oxide via the McCormack reaction.

A standard procedure to form phospholene oxide is McCormac cycloaddition attempted from compound **1a** [10] and dichlorophenylphosphine [11,12]. However, this addition did not give the expected, isolable product. Proposing the disruption of the nitroxide under the reaction conditions applied, we protected nitroxide as an *O*-acetyl derivative **1b** [4]. Treatment of compound **1b** with dichlorophenylphosphine in a three-week-long reaction time in pentane at 37 °C enabled us to obtain compound **2b** after hydrolysis with a modest 36% yield. Deprotection of the *O*-acetyl group by a catalytic amount of NaOMe, followed by oxidation of the *N*-hydroxylamine with MnO_2_, offered 1,1,3,3-tetramethyl-5-phenyl-1,2,3,4,5,6-hexahydrophospholo[3,4-c]pyrrole-5-oxide-2-oxyl **2a** as the first pyrroline nitroxide condensed phospholene oxide (Scheme 1). Compound **2a** was reduced to its hydroxylamine derivative in situ in the NMR tube (see Section 3.1.) and in the resulted ^31^P-NMR we found a single peak at 61.8 ppm and methylene protons as multiplets 2.68–2.73 and 2.85–2.29 ppm with 2H–2H integrals, suggesting that compound **2a** contains an endocyclic double bond.

### 2.2. Synthesis of Pyrroline Nitroxide Diphenylphosphine and Its Phosphonium Salt

**Figure 1:**
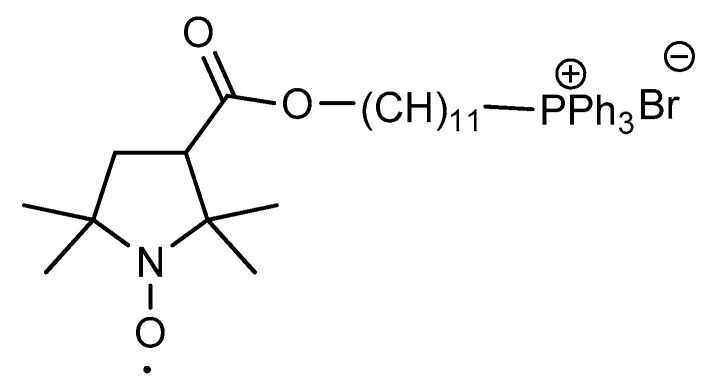
Structure of MITO-CP.

As triphenylphosphine is an essential building block of mitochondria-targeted antioxidants and neoplastic agents [13,14], e.g., lipophilic triphenylphosphonium cations, we aimed to synthesize the paramagnetic analog of triphenylphosphine. We intended to determine whether or not we can synthesize different types of nitroxide-containing mitochondrially targeted molecules compared to MITO-CP (Figure 1) [15]. In our case, nitroxide would function as a superoxide dismutase (SOD) mimic at the cationic “warhead”.

**Scheme 2:**
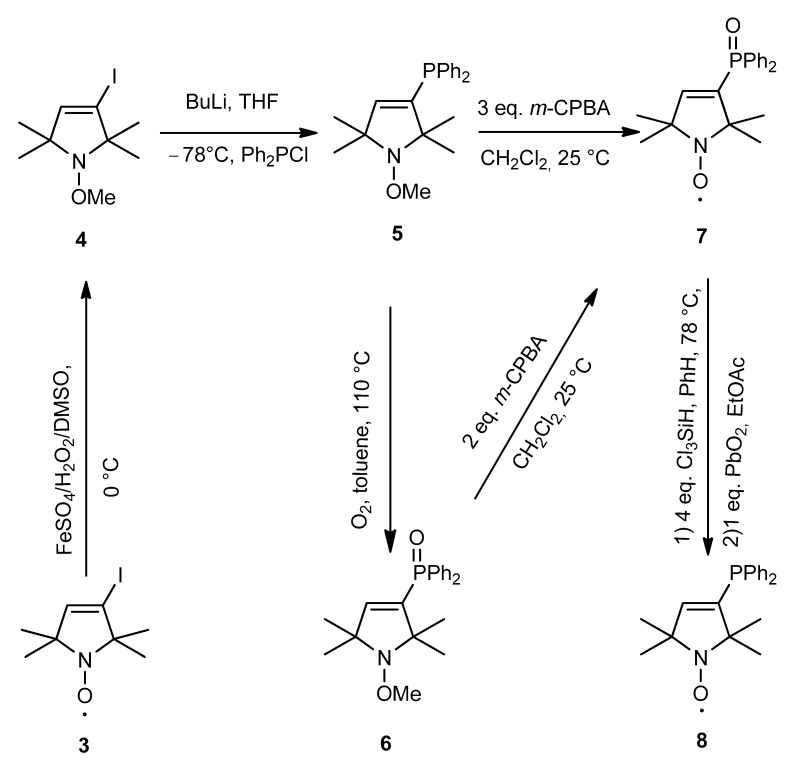
Synthesis of pyrroline nitroxide diphenylphosphine and pyrroline nitroxide diphenylphosphin oxide.

Compound **3** [16] was converted to the corresponding **4** *O*-methyl derivative in a coupled Fenton reaction, which generates methyl radicals. This protected pyrroline nitroxide **4** was treated with 1.1 eq. BuLi followed by the addition of diphenylchlorophosphine to furnish compound **5**. Phosphine **5** proved to be rather stable during the flash chromatography purification process and could be stored for weeks under an Ar atmosphere at −18 °C without oxidation (e.g., appearance of compound **6**). However, refluxing in toluene in air oxidized it to phosphine **6** oxides. Treatment of compounds **5** and **6** with *meta*-chloroperbenzoic acid (*m*-CPBA) to remove the protecting methyl group from the oxygen atom furnished paramagnetic phosphine oxide **7**. Compound **7** could be reduced with paramagnetic pyrroline nitroxide diphenylphospine by heating it with 4 eq. trichlorosilane in toluene at 80 °C to reduce the phosphinoxide function [17,18] to phosphine and reduce nitroxide to hydroxylamine. The latter could be selectively oxidized to nitroxide **8** by PbO_2_ (Scheme 2) without oxidation of phosphorus.

**Scheme 3:**
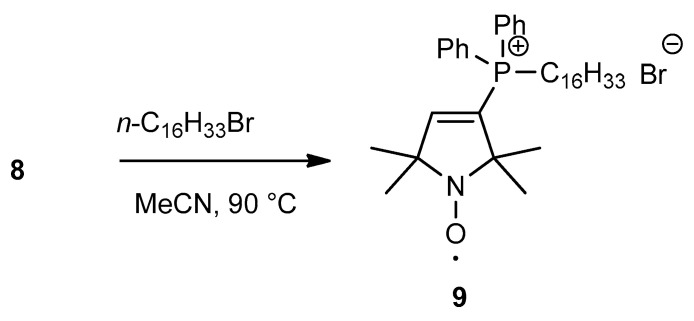
Synthesis of a paramagnetic phosphonium salt.

Compound **8** was heated with hexadecylbromide for 5 days in acetonitrile at 90 °C in a closed vial to afford compound **9** in a low 5% yield in a sluggish reaction, presumably because of sterical hindrance due to the pyrroline nitroxide ring and because of side-reactions (Scheme 3).

### 2.3. X-ray Crystallographic Study of Pyrroline Nitroxide-Diphenylphosphine

**Figure 2:**
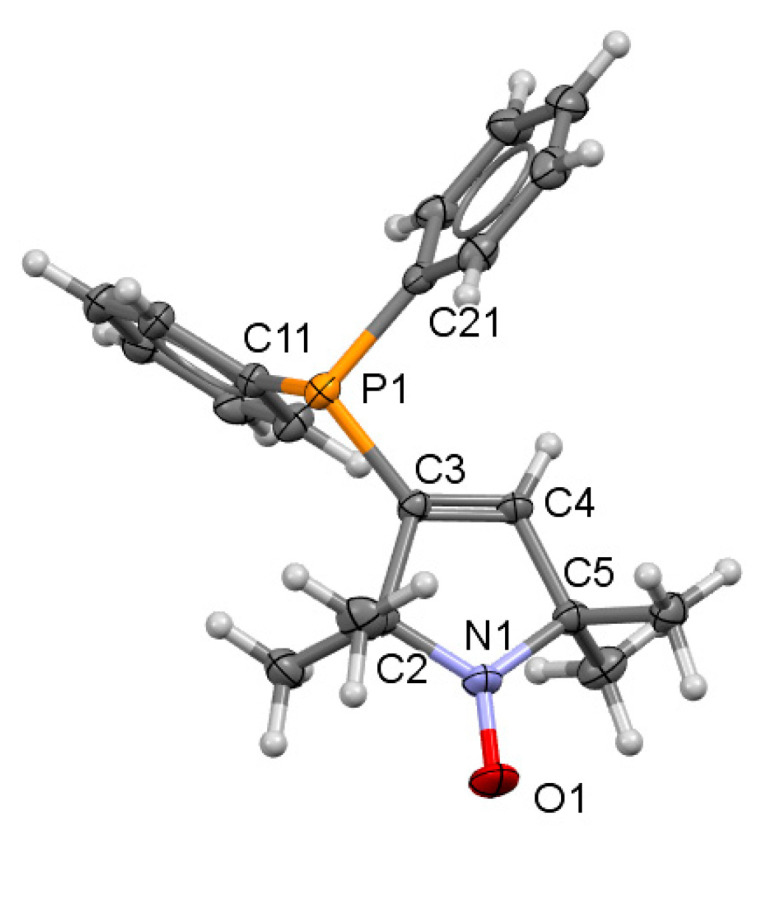
Ortep style view of **8** with a partial numbering scheme showing thermal displacement ellipsoids drawn at the 50% probability level. Key bond lengths [Å] and angles [°] are P1-C3 1.827(2), P1-C21 1.835(2), P1-C11 1.827(2), N1-O1 1.271(3), C3-C4 1.322(3), N1-C2 1.480(3), N1-C5 1.476(3); O1-N1-C5 122.2(2), C5-N1-C2 114.94(18), C4-C3-P1 129.02(18), C2-C3-P1 119.46(17).

X-ray-quality crystals of **8** were grown by slow crystallization from pentane/Et_2_O (2:1) solution by spontaneous evaporation of the solvent. A suitable crystal was fixed under a microscope onto a Mitegen loop using high-density oil. Diffraction intensity data were collected at 200 K using a Bruker-D8 Venture diffractometer (Bruker AXS GmbH, Karlsruhe, Germany) equipped with INCOATEC IμS 3.0 (Incoatec GmbH, Geesthacht, Germany) dual (Cu and Mo) sealed tube micro sources and a Photon II Charge-Integrating Pixel Array detector (Bruker AXS GmbH, Karlsruhe, Germany) using Mo Kα (λ = 0.71073 Å) radiation. High multiplicity data collection and integration were performed using APEX3 (version 2017.3-0, Bruker AXS Inc., 2017, Madison, WI, USA) software. Data reduction and multiscan absorption correction were performed using SAINT (version 8.38A, Bruker AXS Inc., 2017, Madison, WI, USA). The structure was solved using direct methods and refined on F^2^ using the SHELXL program [19] incorporated into the APEX3 suite. Refinement was performed anisotropically for all nonhydrogen atoms. Hydrogen atoms were placed into geometric positions. The CIF file was manually edited using Publcif software [20], while graphics were prepared using the Mercury program [21]. The results for the X-ray diffraction structure determinations were very good according to the Checkcif functionality of PLATON software (Utrecht University, Utrecht, The Netherlands) [22], and structural parameters such as bond length and angle data were in the expected range (for selected data, see the caption for Figure 2). The crystallographic and refinement details are given in Table 1. CCDC contains the supplementary crystallographic data for **8** with deposition number 2082286. These data can be obtained free of charge from The Cambridge Crystallographic Data Centre via www.ccdc.cam.ac.uk/data_request/cif accessed on 21 May 2021.

**Figure 3:**
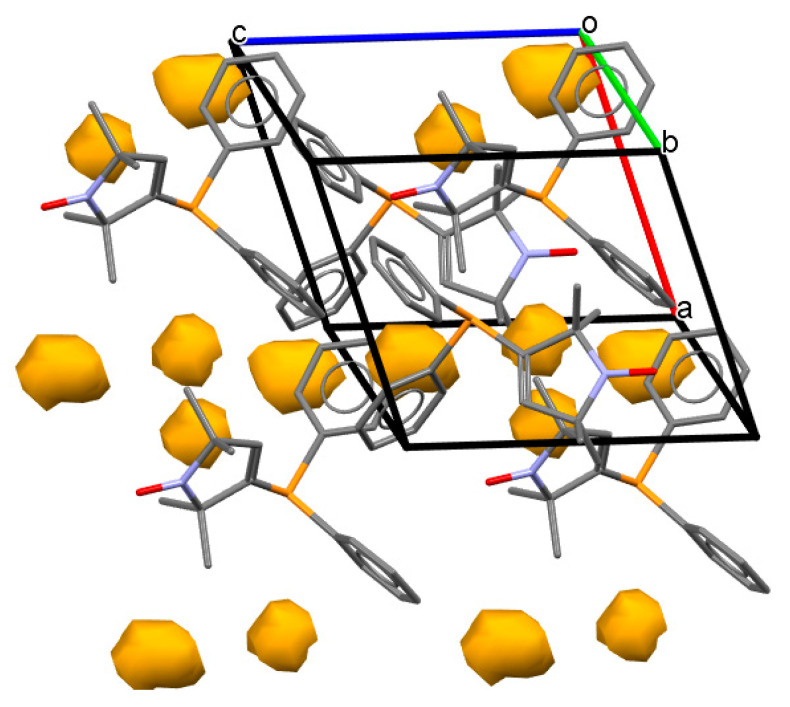
Packing diagram of **8**. Hydrogen atoms are omitted for clarity.

The molecular structure of **8** as the targeted nitroxide derivative of the diphenylphosphinopyrrole derivative is fully supported by the X-ray diffraction study. Both the N-O distance and the double bond between C3 and C4 (see Figure 2) were proven. A search of the Cambridge Structural Database (version 5.41 Updates March 2020) [23] revealed 73 hits for similar 2,2,5,5 tetramethyl pyrrole nitroxide compounds, with an average N-O distance of 1.278(33) Å. We observed a similar value of 1.271(3) Å. However, no phosphorous derivative at C3 or C4 could be found, showing the uniqueness of our compound. The compound crystallized in the monoclinic space group P21, is chiral. Moreover, the Flack parameter is very close to 0 (Table 1) indicating that we have a chiral lattice for our achiral molecule. The packing diagram shows a very small portion of the unit cell as a void (Figure 3).

## 3. Materials and Methods

### 3.1. General Methods and Reagents

The mass spectra were recorded with a GCMS-2020 (Shimadzu, Tokyo, Japan) operated in EI mode (70 eV) and a ThermoScientific Q-Extractive HPLC/MS/MS with ESI(+) ionization (Thermo Scientific, Waltham, MA, USA). Elemental analyses were carried out with a Fisons EA 1110 CHNS elemental analyzer (Fisons Instruments, Milan, Italy). The melting points were determined with a Boetius micromelting point apparatus (Franz Küstner Nachf. K. G., Dresden, Germany). ^1^H NMR spectra were recorded with a Bruker Avance 3 Ascend 500 system (Bruker BioSpin Corp., Karlsruhe, Germany) operated at 500 MHz, and ^13^C NMR spectra were obtained at 125 MHz and ^31^P NMR 202 MHz in CDCl_3_ or DMSO-*d*_6_ at 298 K. The “in situ” reduction of nitroxides was achieved by the addition of five equivalents of hydrazobenzene (DPPH/radical). The EPR spectra were recorded using a MiniScope MS 200 (Magnettech GMBH, Berlin, Germany) in CHCl_3_ solution, and the sample concentrations were 1.0 × 10^−4^ M. All monoradicals gave a triplet line at a_N_= 14.4 G. IR spectra were obtained with a Bruker Alpha FT-IR instrument (Bruker Optics, Ettlingen, Germany) with ATR support on a diamond plate. All spectra are shown in the Supplementary Material. Flash column chromatography was performed using a Merck Kieselgel 60 (0.040–0.063 mm). Qualitative TLC was carried out using commercially available plates (20 × 20 × 0.02 cm) coated with Merck Kieselgel (Darmstadt, Germany) GF_254_. Compounds **1a** [9], **3** [15] were synthesized as previously reported. All the other reagents were purchased from Sigma Aldrich (St. Louis, MO, USA), Molar Chemicals (Halásztelek, Hungary).

### 3.2. Synthesis of 2,2,5,5-Tetramethyl-3,4-dimethylenepyrrolidin-1-yl Acetate (**1b**)

A solution of ascorbic acid (8.80 g, 50.0 mmol) in H_2_O (10 mL) was added to a solution of radical **1a** (1.66 g, 10.0 mmol) in dioxane (30 mL), and the mixture was stirred at 40 °C for 15 min under N_2_. The pale-yellow solution was extracted with CHCl_3_ (2 × 20 mL) and dried on MgSO_4_ under N_2_. Acetyl chloride (860 mg, 10.0 mmol) was added at 0 °C, followed by the slow addition of Et_3_N (1.10 g, 11.0 mmol) at this temperature. Stirring was continued for 1 h at room temperature, and after adding ethanol (1 mL), the reaction mixture was filtered, and the filtrate was evaporated to dryness. The residue was partitioned between brine (15 mL) and EtOAc (20 mL). The organic phase was separated, and the aqueous phase was washed with EtOAc (2 × 10 mL). The combined organic phase was dried (MgSO_4_), filtered, and evaporated, and after flash chromatography purification (hexane-Et_2_O, 6:1), we obtained compound **1b (**1.79 g, 86%) as a white solid, mp 35–37 °C; TLC (hexane/Et_2_O, 5:1): R_f_ = 0.43, IR: 2974, 2933, 1766, 1618 cm^−1^. ^1^H NMR (CDCl_3_) δ: 1.30 (s, 12H, 2 × C(CH_3_)_2_), 2.16 (s, 3H, CH_3_CO), 4.89 (s, 2H, CH_2_), 5.44 (s, 2H, CH_2_), ^13^C NMR (CDCl_3_) δ: 19.1 (COCH_3_), 29.6 (4C, C(CH_3_)_2_), 66.4 (2C, C(CH_3_)_2_), 104.2 (2C, H_2_C=), 151.1 (2C, C=), 171.2 (1C, C=O). MS (EI): *m*/*z* (%): 209 (M^+^, 1), 194 (11), 167 (14), 152 (100), 120 (16), 43 (9). Anal. calcd. for C_12_H_19_NO_2_: C, 68.87; H, 9.15; N, 6.69%; found: C, 68.75; H, 8.95; N, 6.59%.

### 3.3. Synthesis of 1,1,3,3-Tetramethyl-5-oxido-5-phenyl-5,6-dihydrophospholo[3,4-c]pyrrol-2(1H,3H,4H)-yl Acetate (**2b**)

Compound **1b (**1.88 g, 9.0 mmol), Cu(II) stearate (100 mg, 0.15 mmol as polymerization inhibitor), and pentane (10 mL) were added together with dichlorophenyl phosphine (3.22 g, 18.0 mmol) dropwise into a pressure-proof tube equipped with a magnetic stirrer, with stirring at 0 °C. After addition, the tube was capped and stirred at room temperature for 4 days followed by stirring for 17 days at 37 °C. After cooling, the solution was poured into a 250 mL beaker containing a mixture of ice (40 g) and sat. NaHCO_3_ solution (40 mL). The tube was rinsed with NaHCO_3_ solution (10 mL), CHCl_3_ (10 mL) was poured into the beaker, CHCl_3_ (80 mL) was added, and the two-phase system was stirred for 15 min.

After separation of the organic phase, the aqueous phase was washed with CHCl_3_ (2 × 10 mL), and the combined organic phases were dried (MgSO_4_), filtered, and evaporated. The residue was *purified* by flash column chromatography (hexane/EtOAc 2:1 followed by CHCl_3_/Et_2_O 1:4) to give compound **2b** as a white solid (1.07 g, 36%); mp 150–152 °C; TLC (CHC_l3_/MeOH, 58:2): R_f_ =0.41, IR: 2974, 2927, 1767 cm^−1^. ^31^P NMR (CDCl_3_) δ: 61.7; ^1^H NMR (CDCl_3_) δ: 1.29 (s, 12H, 2 × C(C*H*_3_)_2_), 2.18 (s, 3H, C*H*_3_CO), 2.68–2.73 (m, 2H, C*H*_2_), 2.79–2.85 (m, 2H, C*H*_2_), 7.50–7.74 (m, 5H, Ar*H*). ^13^C NMR (CDCl_3_) δ: 19.2. (COCH_3_), 21.7 (2C, C(CH_3_)_2_), 27.1 (2C, C(CH_3_)_2_), 31.2 (*d*, *J* = 65.5 Hz, 2C, CH_2_), 128.9 (*d*, 2C, Ar*C* *J* = 11 Hz), 129.2. (*d*, 2C, *J* = 10.0 Hz, Ar*C*) 132.3 (1C, Ar*C*), 134.4 (*d*, 1C, Ar*C*, *J* = 94 Hz), 139.4 (2C, *C*=, *J* = 11 Hz), 170.9 (1C, *C*=O); MS (EI): *m*/*z* (%): 333 (M^+^, 1), 318 (19), 291 (54), 276 (100), 259 (38). Anal. calcd. for C_18_H_24_NO_3_P: C, 64.85; H, 7.26; N, 4.20 %; found: C, 64.65; H, 7.15; N, 4.17%.

### 3.4. Synthesis of 1,1,3,3-Tetramethyl-5-phenyl-5-oxido-1,2,3,4,5,6-hexahydrophospholo[3,4-c]pyrrole-2-yloxyl Radical (**2a**)

Freshly prepared NaOMe (from 12 mg, 0.52 mmol Na dissolved in 5 mL MeOH) was added to a solution of compound **2b** (999 mg, 3.0 mmol) in MeOH (20 mL), and the mixture was allowed to remain for 2 h at 25 °C. The solvent was evaporated off, the residue was dissolved in sat. aq. NH_4_Cl solution (10 mL) and extracted with CHCl_3_ (2 × 20 mL). The combined organic phase was dried (MgSO_4_), MnO_2_ (86 mg, 1.0 mmol) was added, and O_2_ was bubbled through for 10 min. After filtration, the reaction mixture was evaporated and purified by flash column chromatography (CHCl_3_/Et_2_O, 2:1) to furnish the title compound as a yellow solid (826 mg, 95%); mp 194–197 °C; TLC (CHCl_3_/MeOH, 58:2): R_f_ =0.37, IR: 2974, 2827 cm^−1^. ^31^P NMR (CDCl_3_ + (PhNH)_2_) δ: 61.8; ^1^H NMR (CDCl_3_+(PhNH)_2_) δ: 1.34 (s, 12H, 2 × C(CH_3_)_2_), 2.70 (*dd*, 2H, CH_2_ *J*^1^ = 7.0 Hz, *J*^2^ = 7.0 Hz), 2.88 (t, 2H, CH_2_ *J* = 16 Hz), aromatic protons overlap with (PhNH)_2_ protons. ^13^C NMR (CDCl_3_) δ: 23.7 (2C, C(CH_3_)_2_), 24.5 (2C, C(CH_3_)_2_), 31.2 (*d*, *J* = 67 Hz, 2C, CH_2_), 68.4 (*d*, 2C, C(CH_3_)_2_, *J* = 9 Hz), 128.9 (*d*, 2C, ArC, *J* =11 Hz), 129.3 (2C, ArC), 132.2 (1C, ArC), 134.3 (*d*, 1C, ArC, *J* = 88 Hz), 139.8 (2C, C=, *J* = 11 Hz), MS (EI): *m*/*z* (%): 290 (M^+^, 13), 275 (31), 260 (73), 245 (100). Anal. calcd. for C_16_H_21_NO_2_P: C, 66.19; H, 7.29; N, 4.82%; found: C, 66.05; H, 7.15; N, 4.84%.

### 3.5. Synthesis of 3-Iodo-1-methoxy-2,2,5,5-tetramethyl-2,5-dihydro-1H-pyrrole (**4**)

First, 30% aq. H_2_O_2_ (5 mL) was added dropwise to a stirred solution of compound **3** (2.66 g, 10.0 mmol) and FeSO_4_·7H_2_O (6.9 g, 25.0 mmol) in DMSO (30 mL) at 0 °C, over 2 h. The reaction was monitored by TLC. Upon consumption of the starting material, the reaction mixture was diluted with water (50 mL) and 10% aq. Na_2_SO_3_ (25 mL). The aqueous solution was extracted with Et_2_O (3 × 30 mL). The combined organic phases were dried (MgSO_4_), filtered, and evaporated, and the crude product was purified by flash column chromatography (hexane/Et_2_O, 2:1) to give a colorless oil (2.24 g, 80%); TLC (hexane/Et_2_O, 58:2): R_f_ = 0.52. IR: 2973, 2894, 1610 cm^−1^. ^1^H NMR (CDCl_3_) δ: 1.24 (s, 12H, 2 × C(C*H*_3_)_2_), 3.67 (s, 3H, C*H*_3_CO), 5.94 (s, 1H, *H*C=). ^13^C NMR (CDCl_3_) δ: 22.2 (2C, C(CH_3_)_2_), 29.5 (2C, C(CH_3_)_2_), 64.9 (1C, OCH_3_), 70.7 (1C, *C*(CH_3_)_2_), 72.9 (1C, *C*(CH_3_)_2_), 101.6 (1C, I*C=),* 142.8 (1C, H*C*=). MS (EI): *m*/*z* (%): 281 (M^+^, 5), 266 (97), 139(100), 108 (33), 83(43). Anal. calcd. for C_9_H_16_INO: C, 38.45; H, 5.74; N, 4.98 %; found: C, 38.36; H, 5.65; N, 4.99%.

### 3.6. Synthesis of 3-(Diphenylphosphino)-1-methoxy-2,2,5,5-tetramethyl-2,5-dihydro-1H-pyrrole (**5**)

*n*-BuLi solution in hexane (2.8 mL, 7.0 mmol, 2.5 M) diluted with anhydr. THF (10 mL) was added dropwise to a stirred solution of compound **4** (1.97 g, 7.0 mmol) in anhydr. THF (10 mL) at −78 °C under N_2_. After the addition was completed, the mixture was continuously stirred for 1 h at −78 °C. Then, a solution of diphenylchlorophosphine (1.55 g, 7.0 mmol) in anhydr. THF (10 mL) was added dropwise. After stirring at this temperature for 30 min, the reaction mixture was allowed to warm to r.t. with continuous stirring for 2 h. A sat. aq. NH_4_Cl solution (5 mL) was added, the mixture was extracted with EtOAc (2 × 20 mL), the combined organic phase was dried (MgSO_4_), filtered and evaporated, and the crude product was purified by flash column chromatography (hexane/Et_2_O, 4:1) to give a white powder (1.6 g, 67%); mp 95–97 °C TLC (hexane/Et_2_O, 58:2): R_f_ =0.31. IR: 3056, 2971, 1620, 1572 cm^−1^. ^31^P NMR (CDCl_3_) δ: −27.0; ^1^H NMR (CDCl_3_) δ: 1.26 (s, 12H, 2 × C(CH_3_)_2_), 3.70 (s, 3H, OCH_3_), 5.28 (s, 1H, HC=), 7.37(bs, 10H, ArH). ^13^C NMR (CDCl_3_) δ: 23.0 (2C, C(CH_3_)_2_), 29.6 (2C, C(CH_3_)_2_), 65.0 (1C, OCH_3_), 68.5 (1C, C(CH_3_)_2_), 73.1 (*d*, 1C, C(CH_3_)_2_, *J* =24 Hz), 128.35 (*d*, 4C, ArC, *J* = 7 Hz), 128.7 (2C, ArC), 133.9 (*d*, 4C, ArC, *J* = 20 Hz), 135.8 (*d*, 2C, ArC, *J* = 16 Hz), 143.2 (*d*, 1C, =CP, *J* = 18 Hz), 143.5 (*d*, 1C, HC=, J = 2.6 Hz), MS (EI): *m*/*z* (%): 339 (M^+^, 5), 324 (100), 293 (78), 201 (12), 185 (45), 108 (44). Anal. calcd. for C_21_H_26_NOP: C, 74.31; H, 7.72; N, 4.13%; found: C, 74.21; H, 7.66; N, 4.11%.

### 3.7. Synthesis of 1-Methoxy-2,2,5,5-tetramethyl-2,5-dihydro-1H-pyrrol-3-yl)diphenylphosphine Oxide (**6**)

A solution of compound **5** (679 mg, 2.0 mmol) in toluene (15 mL) was heated at the reflux temperature for 12 h. The solvent was evaporated, and the crude material was subjected to flash column chromatography (hexane/EtOAc, 2:1) to give a yellow powder (355 mg, 50%); mp 128–130 °C; TLC (CHCl_3_/Et_2_O, 2:1): Rf =0.46. IR: 2973, 2674, 1606 cm^−1^. ^31^P NMR (CDCl_3_) δ: 23.5; ^1^H NMR (CDCl_3_) δ: 1.28 (s, 6H, C(CH_3_)_2_), 1.36 (s, 6H, C(CH_3_)_2_), 3.65 (s, 3H, OCH_3_), 5.68 (*d*, 1H, HC=, *J* = 11.5 Hz), 7.48–7.72 (m, 10H, ArH). ^13^C NMR (CDCl_3_) δ: 22.7 (2C, C(CH_3_)_2_), 29.7 (2C, C(CH_3_)_2_), 65.1 (1C, OCH_3_), 68.8 (*d*, 1C, C(CH_3_)_2_, *J* = 13 Hz), 73.2 (*d*, 1C, C(CH_3_)_2_, *J* =11 Hz), 128.4 (*d*, 4C, ArC, *J*.=12 Hz), 131.75 (2C, ArC), 131.79 (*d*, 4C, ArC *J* = 4 Hz), 132.3 (*d*, 2C, ArC, *J* = 124 Hz), 138.6 (*d*, 1C, PC=, *J* = 101 Hz), 150.1 (*d*, 1C, =CH, *J* = 8 Hz). MS (EI): *m*/*z* (%): 355 (M^+^, 2), 340 (90), 310 (10), 308 (45), 201 (100), 108 (25). Anal. calcd. for C_21_H_26_NO_2_P: C, 70.97; H, 7.37; N, 3.94%; found: C, 71.07; H, 7.41; N, 3.87%.

### 3.8. Synthesis of 3-(Diphenylphosphinoxido)-2,2,5,5-tetramethyl-2,5-dihydro-1H-pyrrol-1-yloxyl Radical (**7**)

First, 3-chloroperbenzoic acid (3.0 eq. for compound **5**, 2.0 eq. for compound **6**) was added in 2–3 portions at 0 °C to a stirred solution of compound **5** or **6** (2.0 mmol) in anhydr. CH_2_Cl_2_ (5 mL) for 10 min. The solution was stirred for an additional 30 min at ambient temperature. Then, the solution was washed with 10% aq. Na_2_CO_3_ solution (2 × 10 mL), and the organic phase was separated, dried (MgSO_4_), filtered, and evaporated. The residue was subjected to flash column chromatography (hexan-EtOAc, 1:9) to give a yellow powder (612 mg, 90%); mp 135–137 °C; TLC (CHCl_3_/MeOH, 58:2): R_f_ =0.56. IR: 2973, 2925, 1596 cm^−1^. ^31^P NMR (CDCl_3_+(PhNH)_2_) δ: 23.4; ^1^H NMR (CDCl_3_+(PhNH)_2_) δ: 1.32 (s, 6H, C(CH_3_)_2_), 1.42 (s, 6H, C(CH_3_)_2_), 5.82 (*d*, 1H, HC=, *J* = 11.5 Hz), aromatic protons overlap with (PhNH)_2_ signals. ^13^C NMR (CDCl_3_) δ: 25.1 (2C, C(CH_3_)_2_), 25.8 (2C, C(CH_3_)_2_), 69.1 (*d*, 1C, C(CH_3_)_2,_ *J* = 12 Hz), 73.4 (*d*, 1C, C(CH_3_)_2_, *J* =11 Hz), 128.5 (*d*, 4C, ArC, *J* = 12 Hz), 131.8 (*d*, 4C, ArC, *J* = 10 Hz), 131.90 (s, 2C, ArC), 132.6 (*d*, 2C, ArC, *J* = 4 Hz), 138.9 (*d*, 1C, PC=, *J* = 101 Hz), 149.8 (*d*, 1C, =CH, *J* = 8 Hz). MS (EI): *m*/*z* (%): 340 (M^+^, 92), 310 (10), 308 (45), 201 (100), 108 (20). Anal. calcd. for C_20_H_23_NO_2_P: C, 70.57; H, 6.81; N, 4.12%; found: C, 70.71; H, 6.77; N, 4.01%.

### 3.9. Synthesis of 3-(Diphenylphosphino)-2,2,5,5-tetramethyl-2,5-dihydro-1H-pyrrol-1-yloxyl Radical (**8**)

Trichlorosilane (0.8 mL, 4 eq., 8 mmol) was added to a stirred solution of compound **7** (680 mg, 2.0 mmol) in anhydr. toluene (10 mL), at 0 °C under N_2._ The resulting mixture was stirred at 80 °C for 4 h under N_2,_ cooled to room temperature and then poured into a 250 mL beaker containing ice (40 g) and 5% aq. NaOH solution (10 mL). The organic phase was extracted with EtOAc (2 × 15 mL), dried (MgSO_4_), oxidized by adding PbO_2_ (478 mg, 2.0 mmol), filtered, and then evaporated. The residue was purified by flash column chromatography (hexane/Et_2_O) to give a yellow powder (421 mg, 65%); mp 120–122 °C; TLC (hexane/Et_2_O, 2:1): R_f_ =0.42. IR: 3054, 2974, 2855, 1598, 1582 cm^−1^. ^31^P NMR (CDCl_3_+(PhNH)_2_) δ: −26.6; ^1^H NMR (CDCl_3_+(PhNH)_2_) δ: 1.32 (s, 6H, C(CH_3_)_2_), 1.34 (s, 6H, C(CH_3_)_2_), 5.45 (*d*, 1H, HC=, *J* = 4 Hz), aromatic protons overlap with (PhNH)_2_ signals. ^13^C NMR (CDCl_3_) δ: 25.1 (2C, C(CH_3_)_2_), 25.8 (2C, C(CH_3_)_2_), 69.1 (*d*, 1C, C(CH_3_)_2,_ *J* = 12 Hz) 73.4 (*d*, 1C, C(CH_3_)_2_, *J* =11 Hz), 128.4 (*d*, 4C, ArC, *J* = 7 Hz), 128.9 (2C, ArC), 131.8 (*d*, 2C, ArC, *J* =10 Hz), 134.0 (*d*, 4C, ArC, *J* = 20 Hz), 135.7 (*d*, 1C, PC=, *J* = 8 Hz), 143.3 (*d*, 1C, =CH, *J* = 2 Hz). MS (EI): *m*/*z* (%): 324 (M^+^, 17), 294 (99), 279 (100), 183 (62), 108 (84). Anal. calcd. for C_20_H_23_NOP: C, 74.05; H, 7.15; N, 4.32%; found: C, 74.21; H, 6.94; N, 4.46%.

### 3.10. Synthesis of Hexadecyl (1-oxyl-2,2,5,5-tetramethyl-2,5-dihydro-1H-pyrrol-3-yl)diphenylphosphonium Bromide Radical (**9**)

A mixture of compound **8** (324 mg, 1.0 mmol) and hexadecylbromide (305 mg, 1.0 mmol) in acetonitrile (10 mL) in a pressure-proof closed vial was stirred and heated at 90 °C for 5 days. After cooling to room temperature, the solvent was evaporated, and the crude material was purified by flash column chromatography (CHCl_3_/MeOH, 9:1) to furnish the title compound as a beige solid (31 mg, 5%); mp 118–120 °C; TLC (CHCl_3_/MeOH, 9:1): R_f_ = 0.40, IR: 2923, 2852, 1598 cm^−1^. ^1^H and ^13^C NMR data were not obtained because of the low solubility of the title compound; high-resolution MS (ESI): *m*/*z* [M]^+^ calc. for C_36_H_56_NOP^+^: 549.4094; found: 549.4099. Anal. calcd. for C_36_H_56_BrNOP: C, 68.66; H, 8.96; N, 2.22%; found: C, 68.47; H, 8.89; N, 2.13%.

## 4. Conclusions

In this paper, we demonstrated that an *O*-acetyl nitroxide-protected paramagnetic diene can be used to synthesize phospholo[3,4-c] pyrrole scaffolds. Protected *O*-methyl pyrroline vinyl iodide can be converted to pyrroline nitroxide-diphenylphosphine oxide, for which reduction afforded pyrroline nitroxide-diphenylphosphine. A single-crystal X-ray diffraction study unambiguously supported the molecular structure. The pyrroline nitroxide diphenylphosphine can be converted into a hexadecyl phosphonium salt. In general, the proposed and adopted approaches could be used for a pyrroline nitroxide-containing phosphine- and pyrroline nitroxide-condensed *P*-heterocycle. Further synthetic and biological study of these compounds are in progress in our laboratory.

## Figures and Tables

**Table 1:** Crystal data for compound **8**.

Chemical Formula	C_20_H_23_NOP
M_r_	324.36
Crystal system, space group	Monoclinic, *P*2_1_
Temperature (K)	200
*a*, *b*, *c* (Å)	8.3032 (3), 12.1786 (4), 9.3477 (3)
β (°)	108.792 (2)
*V* (Å^3^)	894.86 (5)
*Z*	2
Radiation type	Mo *K*α
μ (mm^−1^)	0.16
Crystal size (mm)	0.45 × 0.44 × 0.28
Data collection	
Diffractometer	Bruker D8 VENTURE
Absorption correction	Multiscan *SADABS2016*/2-Bruker AXS area detector scaling and absorption correction
*T*_min_, *T*_max_	0.85, 0.96
No. of measured, independent, and observed (*I* > 2σ(*I*)) reflections	13,054, 3528, 3427
*R*_int_	0.037
(sin θ/λ)_max_ (Å^−1^)	0.619
Refinement	
*R*(*F*^2^ > 2σ(*F*^2^)), *wR*(*F*^2^), *S*	0.031, 0.096, 1.21
No. of reflections	3528
No. of parameters	213
No. of restraints	1
H-atom treatment	H-atom parameters constrained
Δ>_max_, Δ>_min_ (e Å^−3^)	0.51, −0.50
Absolute structure	Flack x determined using 1581 quotients [(I^+^) − (I^−^)]/[(I^+^) + (I^−^)] (Parsons, Flack and Wagner, Acta Cryst. B69 (2013) 249–259).
Absolute structure parameter	–0.05 (3)

## Data Availability

All data generated or analyzed during this study are included in this published article and CCDC contains the supplementary crystallographic data for **8** with deposition number 2082286. These data can be obtained free of charge from The Cambridge Crystallographic Data Centre via www.ccdc.cam.ac.uk/data_request/cif accessed on 21 May 2021.

## Supplementary Materials

Supplementary materials are available online. The ^1^H NMR and ^13^C NMR, IR, MS spectra of novel compounds, and EPR spectra of compound **9** are available online. Table S1: Elememtal analysis of new compounds synthesized, Figure S1: Preliminary biological data of compound **9** and cetyltriphenylphosphonium bromide (compound **10**) compared to MITO-CP.

## Sample Availability

Samples of compounds **2a, 8,** and **9** are available from the authors.
