# Supplementary material for: Syntheses and Study of a Pyrroline Nitroxide Condensed Phospholene Oxide and a Pyrroline Nitroxide Attached Diphenylphosphine
Source: Molecules. 2021 Jul 19;26(14):4366. doi: 10.3390/molecules26144366 (PMC8305133; doi:10.3390/molecules26144366)
Supplement: Supplementary file 1 [file molecules-26-04366-s001.zip › molecules-1249283-supplementary.pdf]

SUPPLEMENTARY MATERIAL

Syntheses and study of a pyrroline nitroxide condensed phospholene oxide and a pyrroline nitroxide attached diphenylphosphine

Mostafa Isbera [1], Balázs Bognár[1], Ferenc Gallyas,[2,3,6] Attila Bényei[4], József Jekő[5], and Tamás Kálai [1,6*]

[1]Institute of Organic and Medicinal Chemistry, Faculty of Pharmacy; University of Pécs, Szigeti st. 12, H-7624 Pécs, Hungary; mostafaisbera@gmail.com balazs.bognar@aok.pte.hu, tamas.kalai@aok.pte.hu
[2]Department of Biochemistry and Medical Chemistry, University of Pécs Medical School, 7624 Pécs, Hungary; ferenc.gallyas@aok.pte.hu
[3]HAS-UP Nuclear-Mitochondrial Interactions Research Group, 1245 Budapest, Hungary
[4]Department of Pharmaceutical Chemistry, University of Debrecen, Egyetem tér 1, H-4032 Debrecen, Hungary; benyei.attila@science.unideb.hu
[5]Department of Chemistry, University of Nyíregyháza, Sóstói st. 31/B, 4440 Nyíregyháza, Hungary; jjozsi@gmail.com
[6]János Szentágothai Research Center, University of Pécs, Ifjúság 20, H-7624 Pécs, Hungary
*Correspondence: tamas.kalai@aok.pte.hu; Tel.: (+3672536221)

Contents

^{1}H NMR, ^{13}C NMR, IR of compound **1b**	3
^{1}H NMR, ^{31}P NMR, ^{13}C NMR, IR of compound **2b**	5
^{1}H NMR, ^{31}P NMR, ^{13}C NMR, IR of compound **2a**	7
^{1}H NMR, ^{13}C NMR, IR of compound **4**	9
^{1}H NMR, ^{31}P NMR, ^{13}C NMR, IR of compound **5**	11
^{1}H NMR, ^{31}P NMR, ^{13}C NMR, IR of compound **6**	13
^{1}H NMR, ^{31}P NMR, ^{13}C NMR, IR of compound **7**	15
^{1}H NMR, ^{31}P NMR, ^{13}C NMR, IR of compound **8**	17
IR and EPR of compound **9**	19
Mass spectra of compounds **1b-9**	20
Elemental analysis of compoumds **1b-9**	25
Preliminary biological data	26

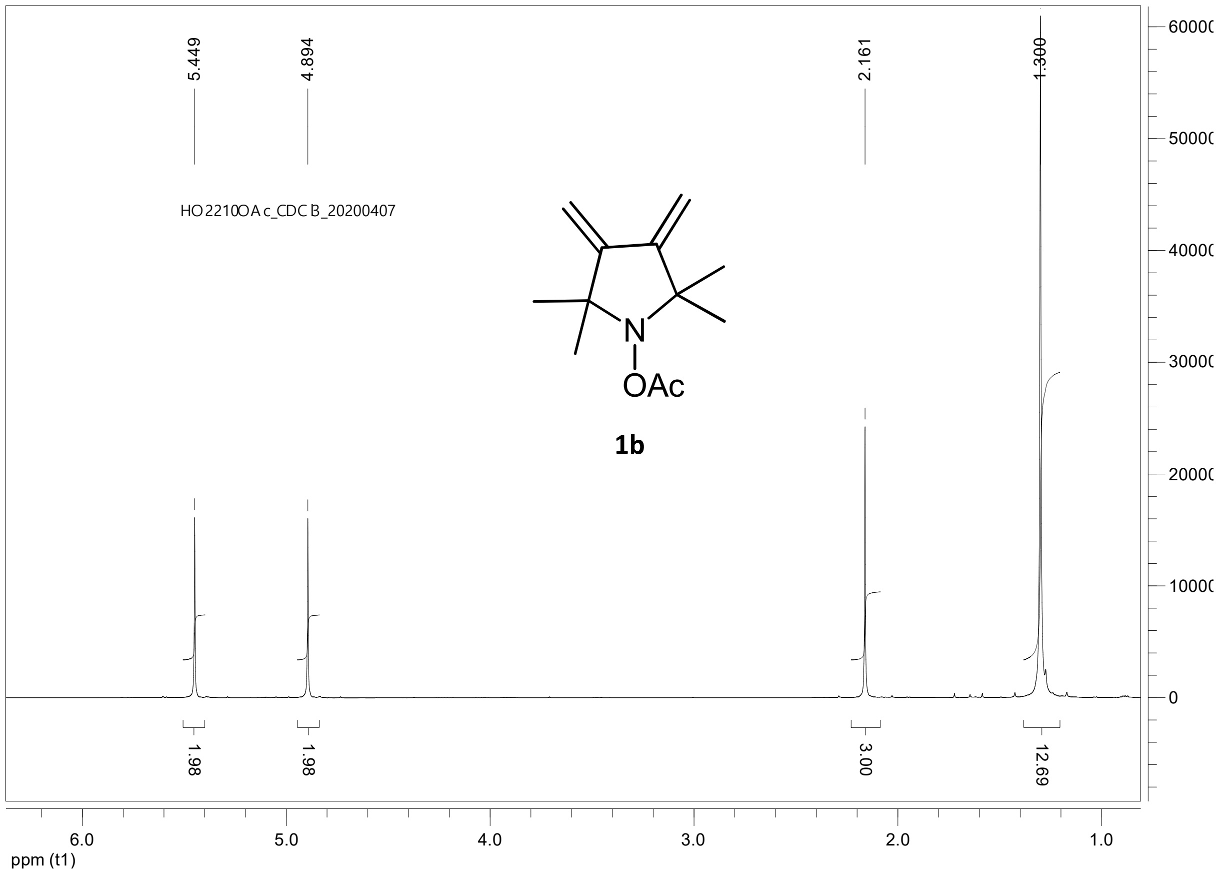
5.449
4.894
2.161
1.300
HO2210OAc_CDCl3_20200407
OAc
N
1b
1.98
1.98
3.00
12.69
ppm (t1)

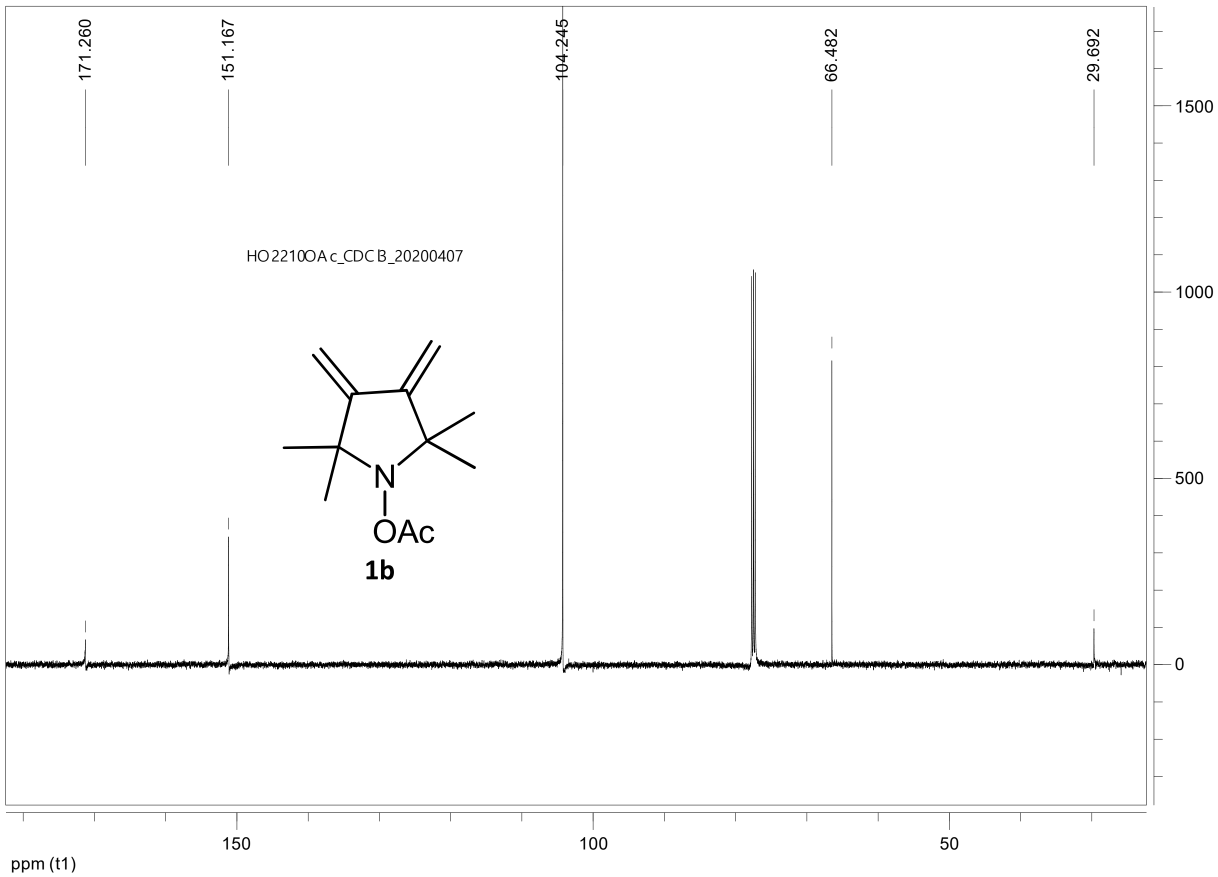
171.260
151.167
104.245
66.482
29.692
HO2210OAc_CDCl3_20200407
N
OAc
1b
ppm (t1)

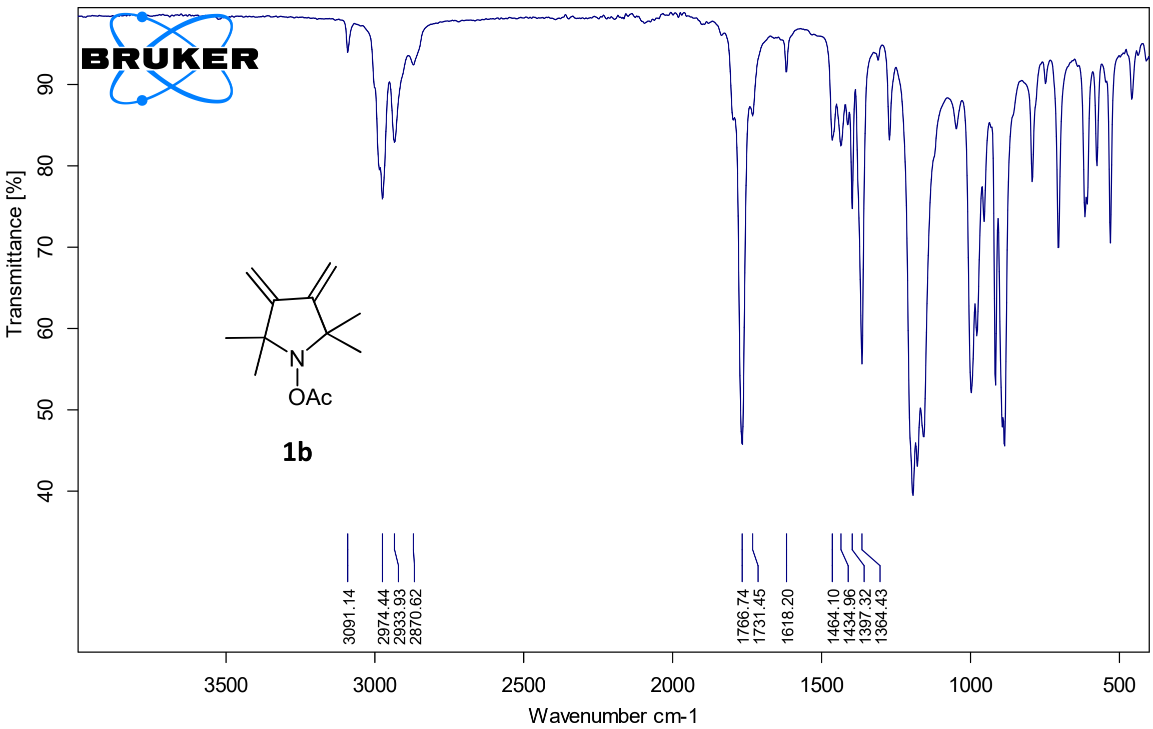

C:\OPUS_7.2.139.1294\Measure\ATR_Di\HO-2210-OAc.0	HO-2210-OAc	Instrument type and / or accessory	2021.04.01

Page 1/1

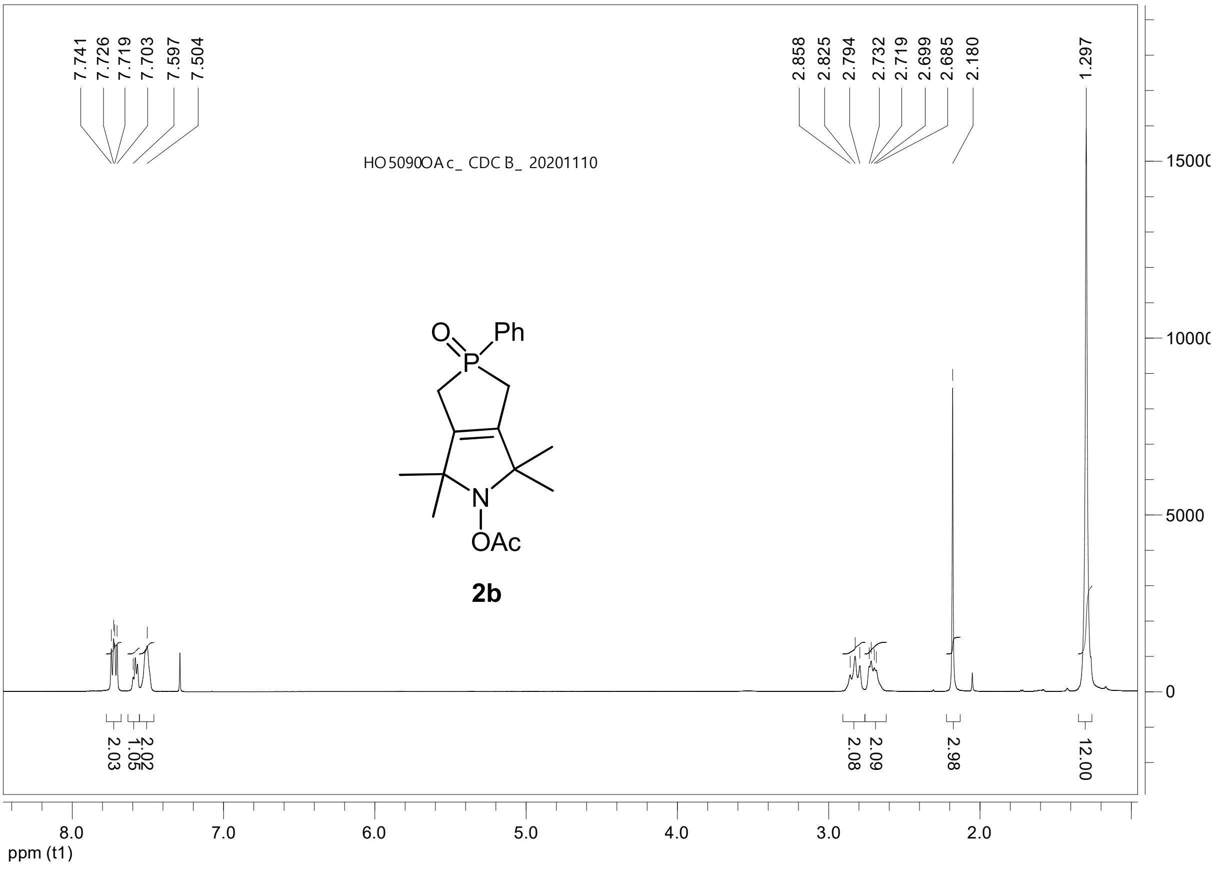
7.741
7.726
7.719
7.703
7.597
7.504
2.858
2.825
2.794
2.732
2.719
2.699
2.685
2.180
1.297
HO5090OAc_ CDCB_ 20201110
O
Ph
P
N
OAc
2b
2.03
1.05
2.02
2.08
2.09
2.98
12.00
8.0
7.0
6.0
5.0
4.0
3.0
2.0
ppm (t1)

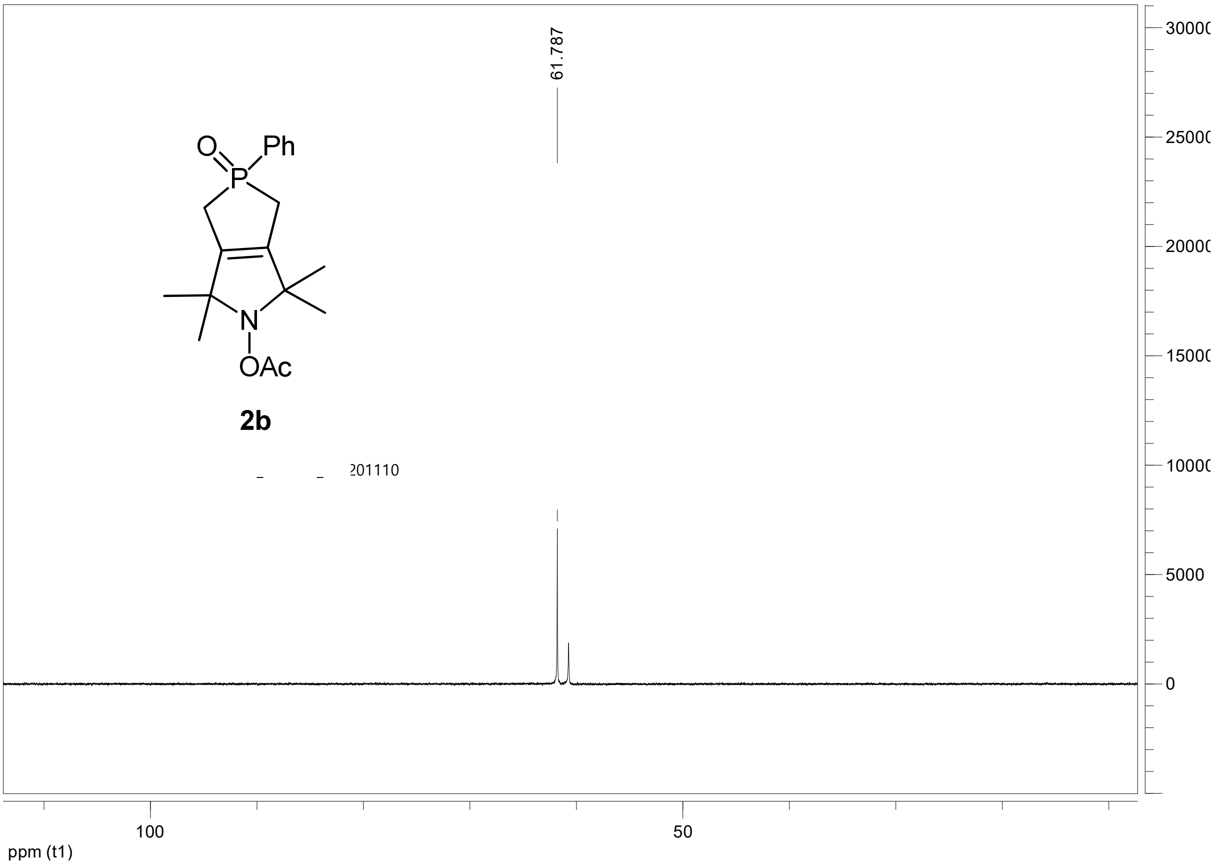
61.787
O
Ph
P
N
OAc
2b
201110
100
50
ppm (t1)

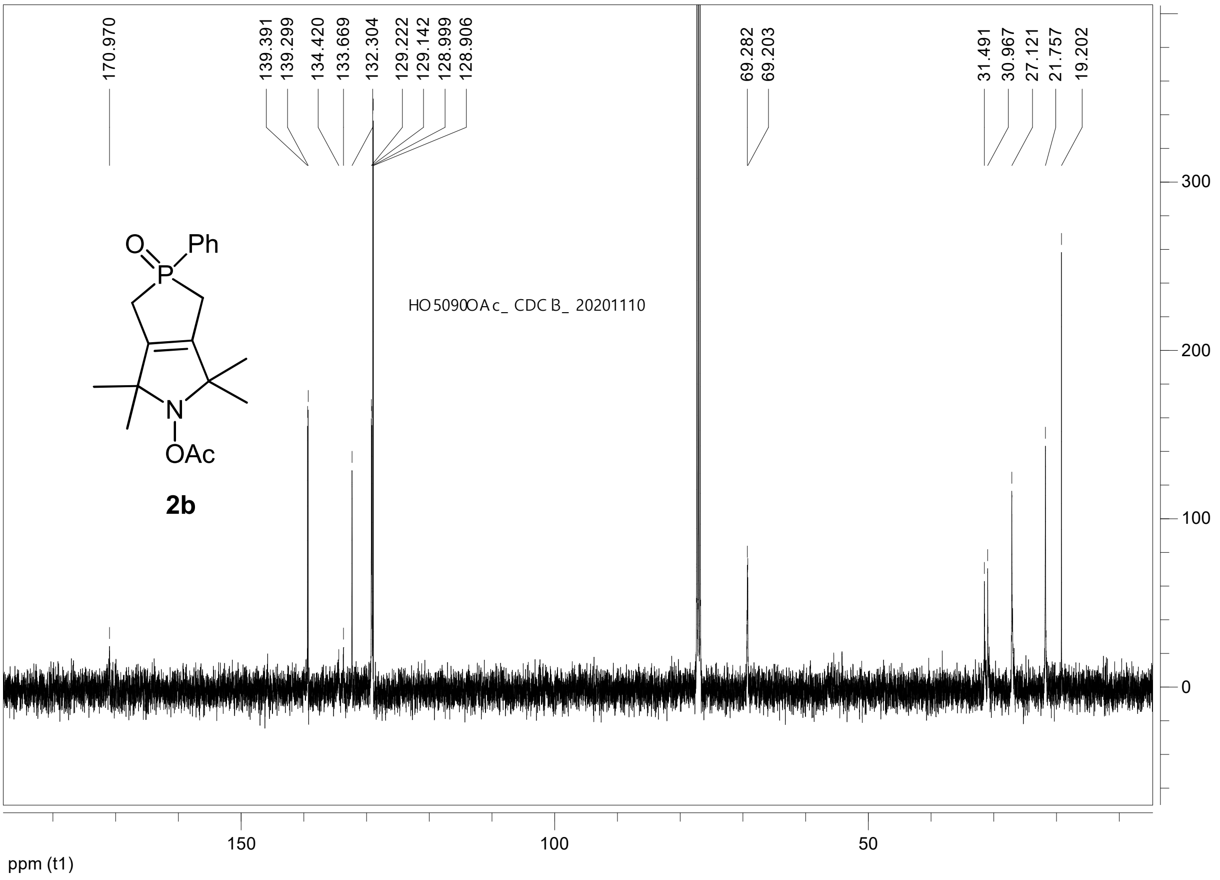

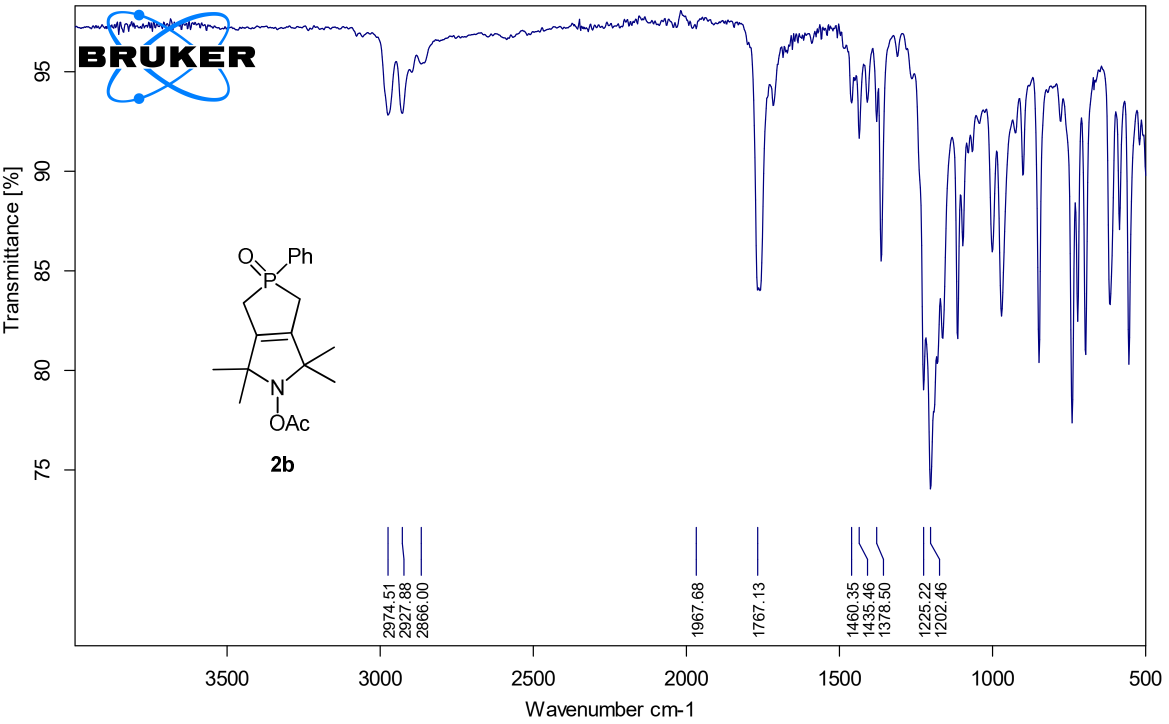

C:\OPUS_7.2.139.1294\Measure\HO-5090 OAc.0	HO-5090 OAc	Instrument type and / or accessory		2020.11.20

Page 1/1

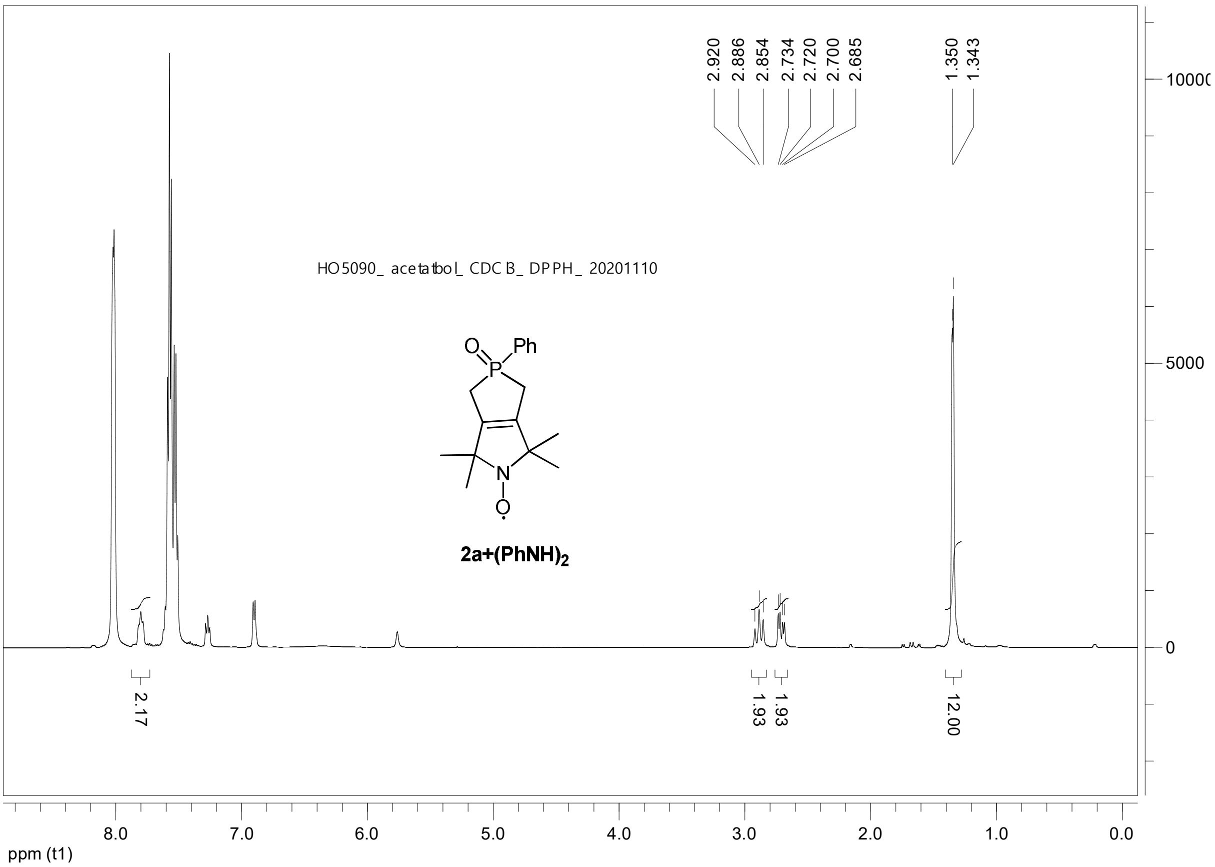
2.920
2.886
2.854
2.734
2.720
2.700
2.685
1.350
1.343
HO5090_ acetatbol_ CDCB_ DPPH_ 20201110
O
Ph
P
N
O
2a+(PhNH)2
2.17
1.93
1.93
12.00
10000
5000
0
8.0
7.0
6.0
5.0
4.0
3.0
2.0
1.0
0.0
ppm (t1)

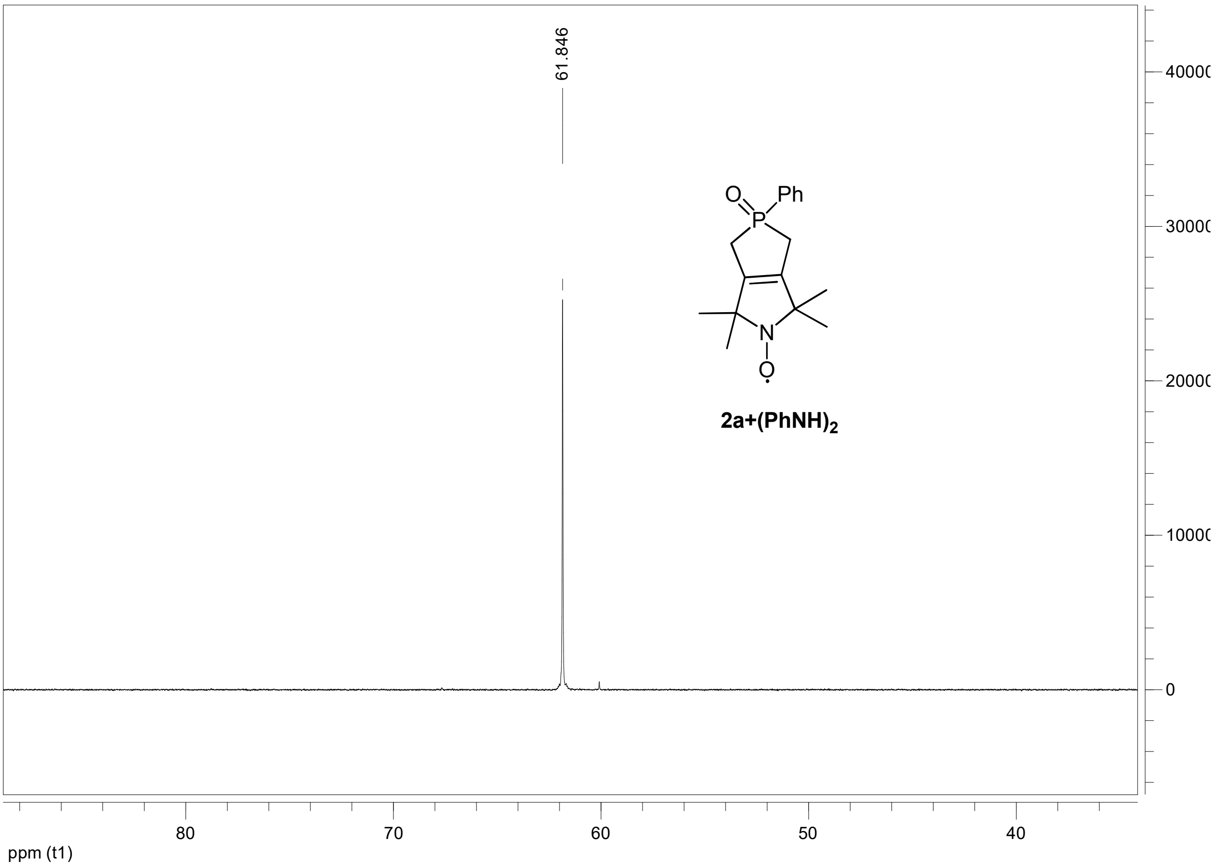
61.846
O
Ph
P
N
O
2a+(PhNH)2
40000
30000
20000
10000
0
80
70
60
50
40
ppm (t1)

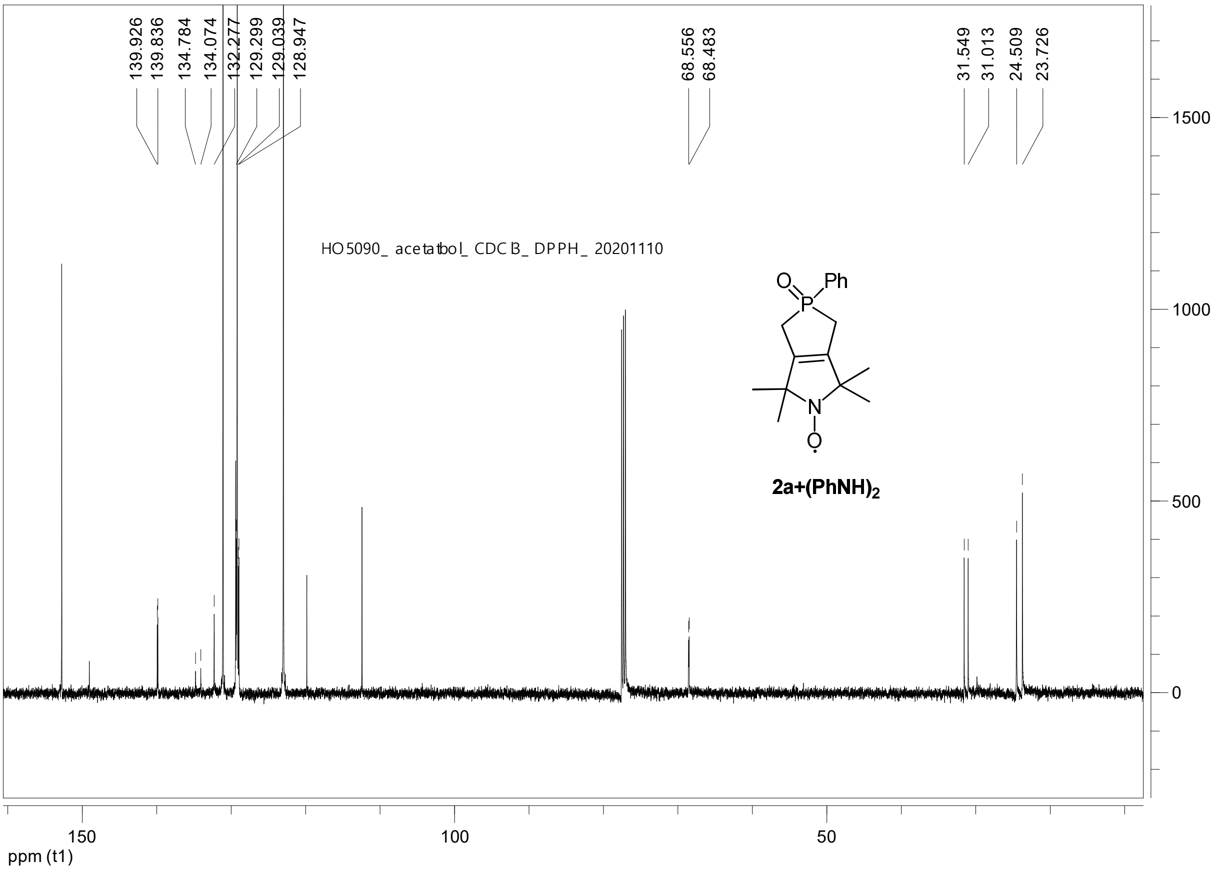

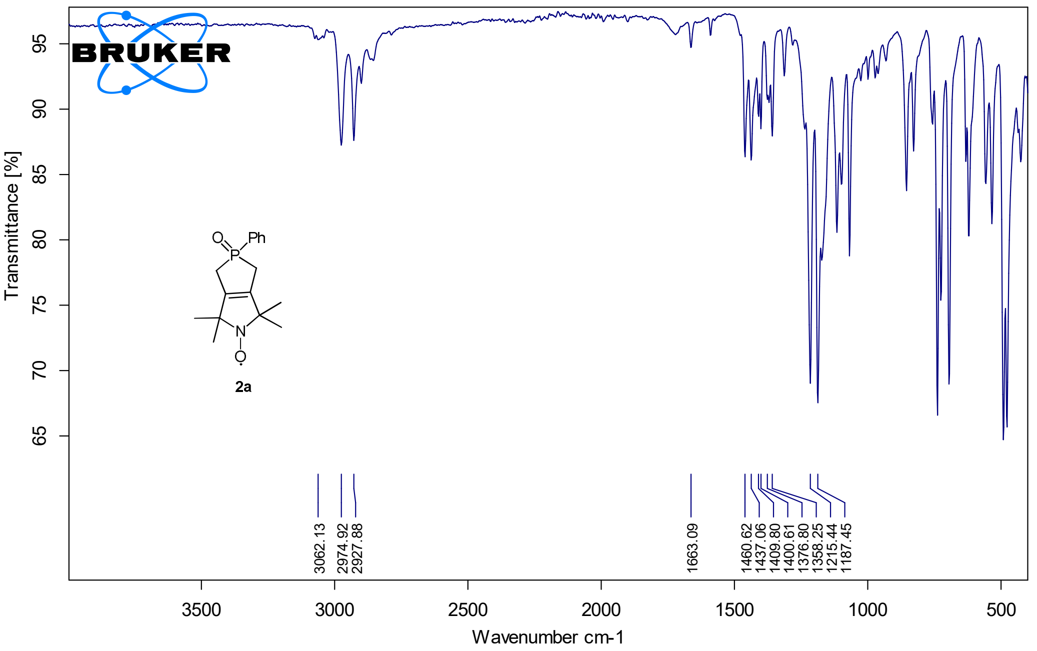

C:\OPUS_7.2.139.1294\Measure\ATR_Di\HO-5090 new.1	HO-5090 new	Instrument type and / or accessory		2021.03.26

Page 1/1

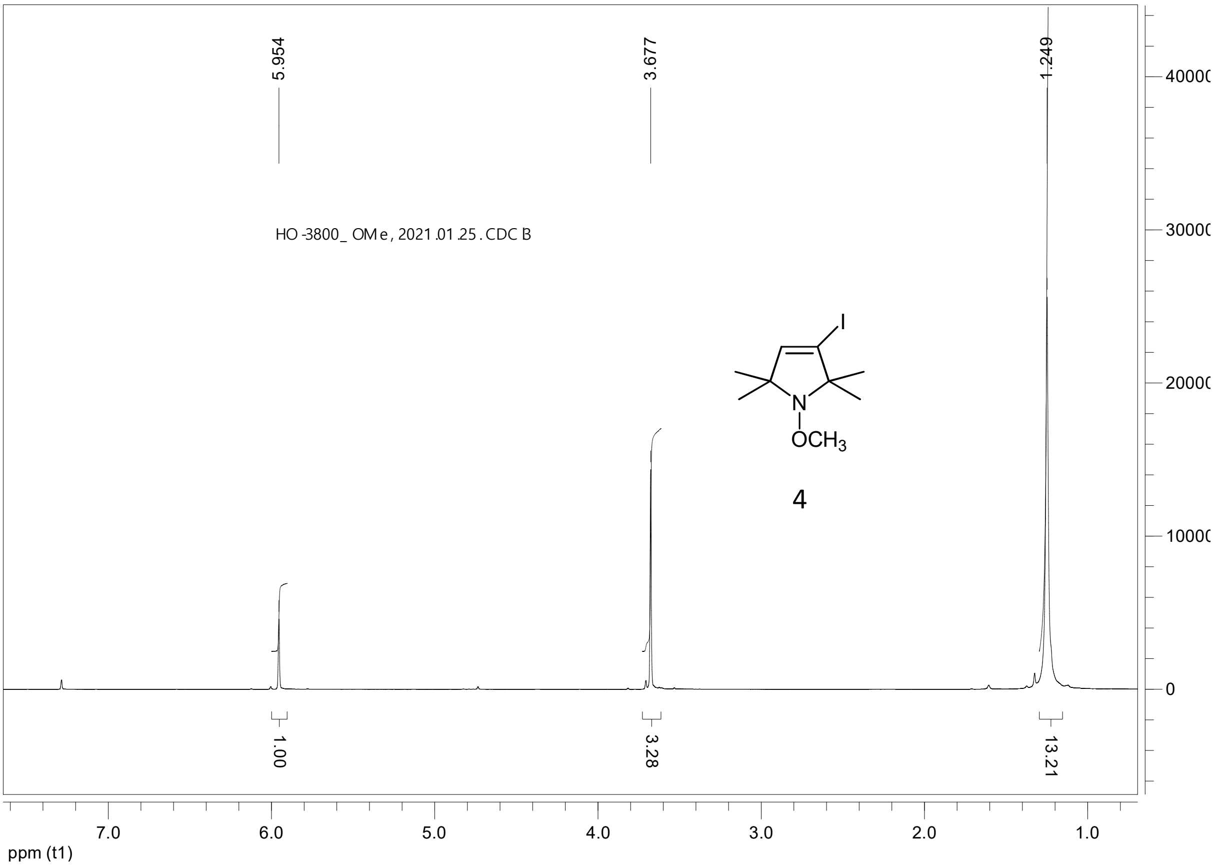
5.954
3.677
1.249
HO -3800_ OMe, 2021.01.25. CDC B
I
N
OCH3
4
1.00
3.28
13.21
40000
30000
20000
10000
0
7.0
6.0
5.0
4.0
3.0
2.0
1.0
ppm (t1)

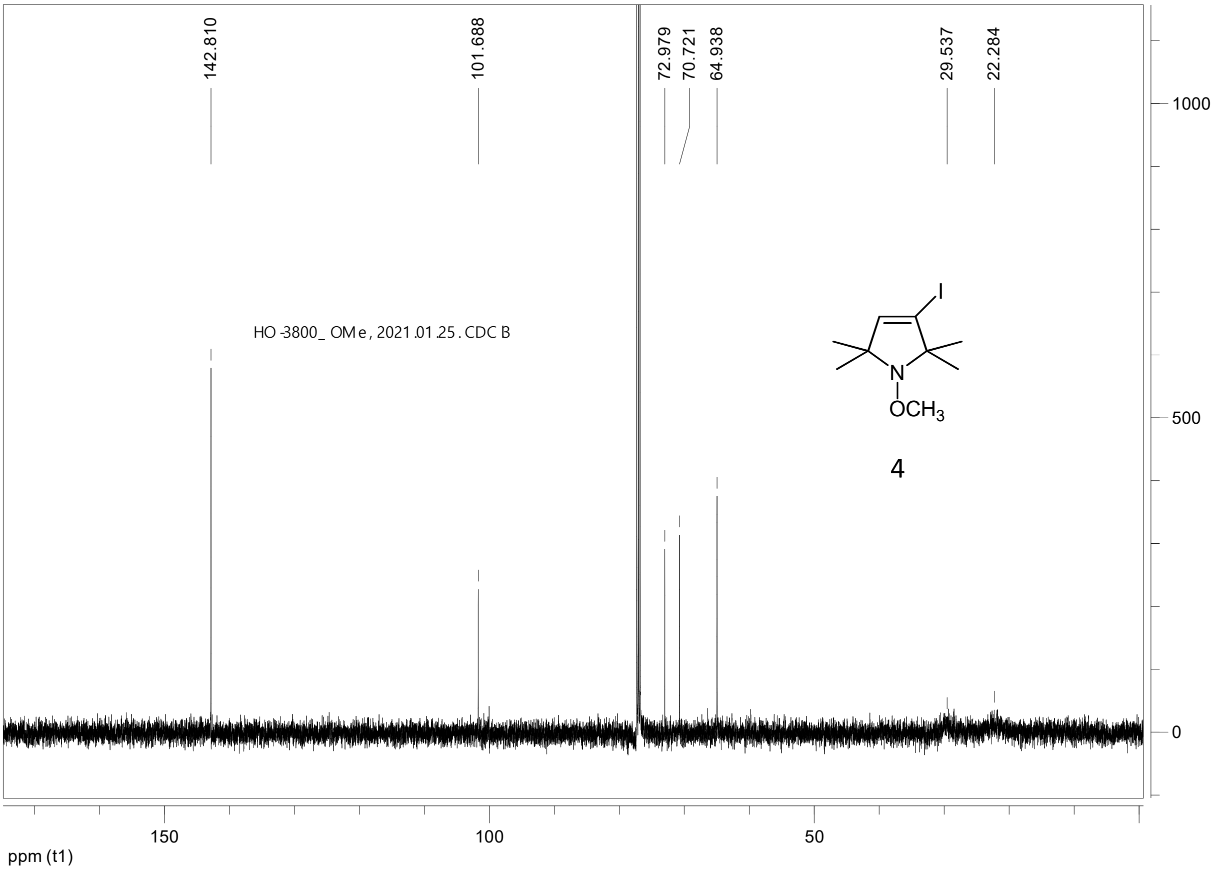
142.810
101.688
72.979
70.721
64.938
29.537
22.284
HO -3800_ OMe, 2021.01.25. CDC B
I
N
OCH3
4
1000
500
0
150
100
50
ppm (t1)

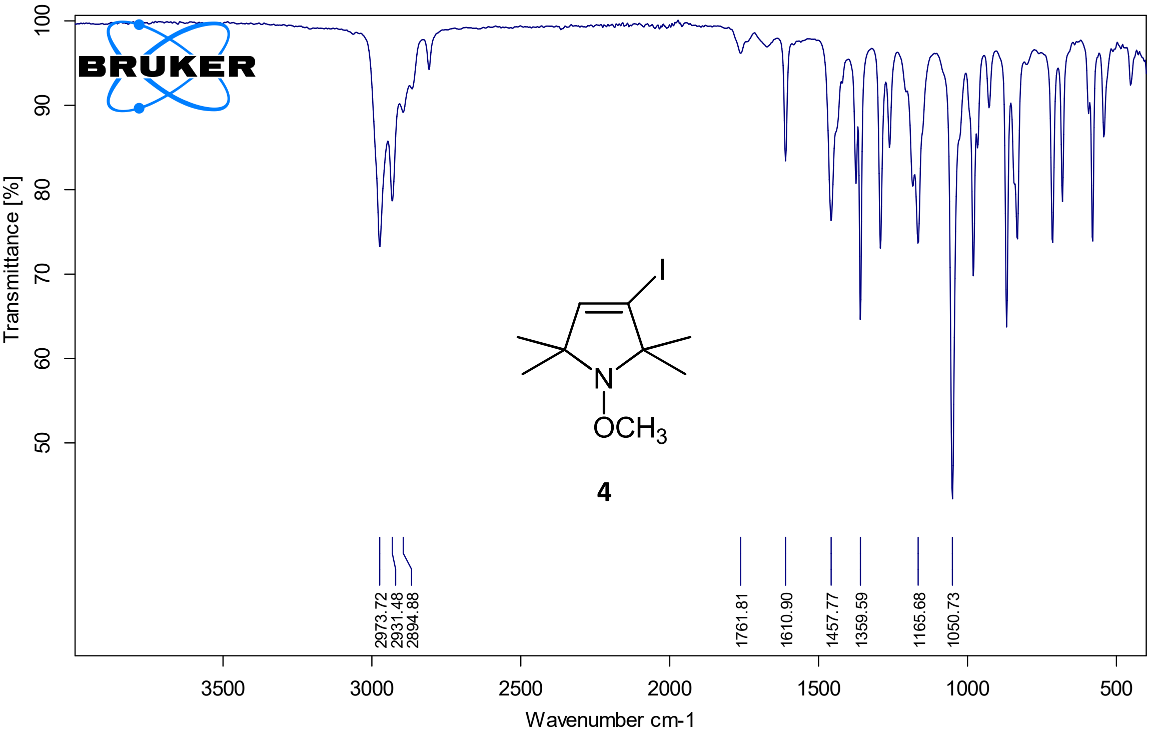

C:\OPUS_7.2.139.1294\Measure\ATR_Di\HO-3800-OMe.0	HO-3800-OMe	Instrument type and / or accessory	2021.03.26

Page 1/1

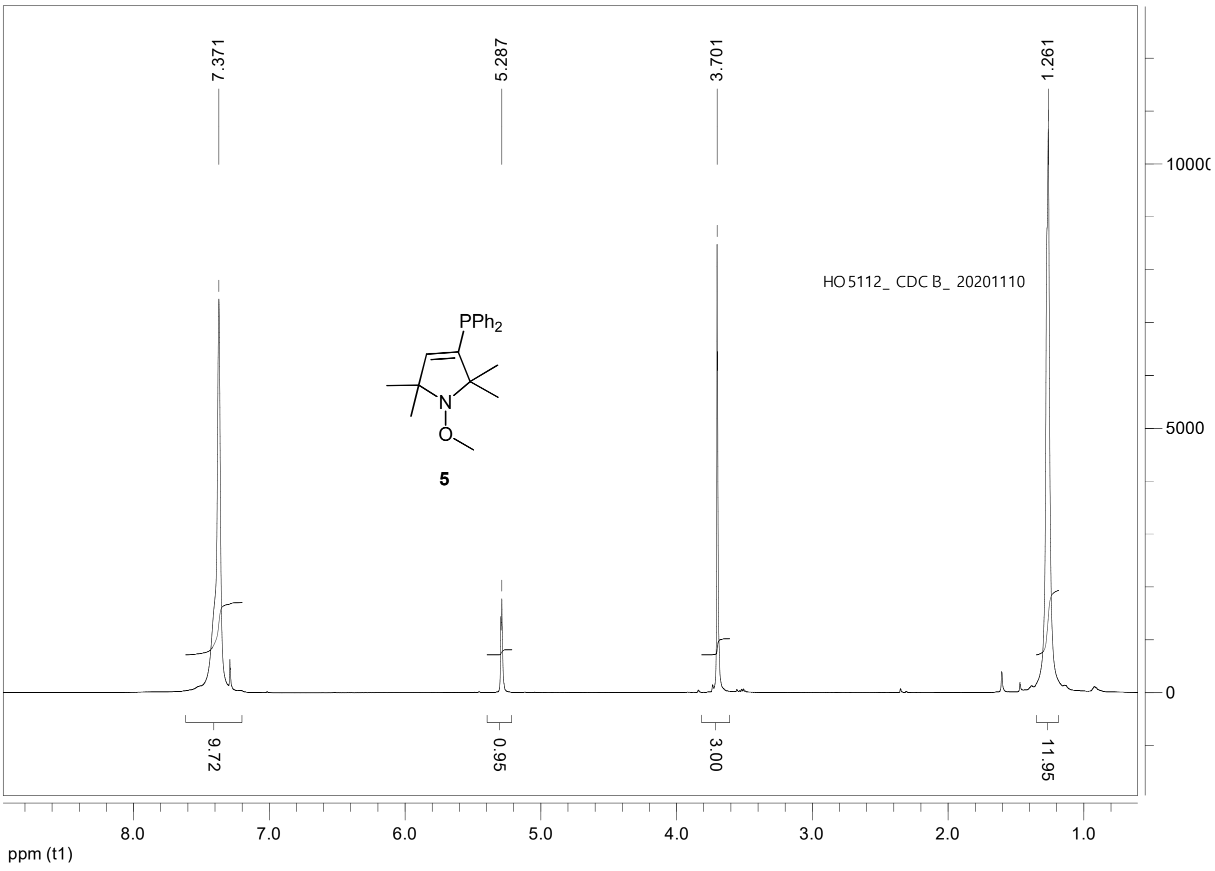

7.371
5.287
3.701
1.261
HO5112_ CDC B_ 20201110
PPh2
N
O
5
9.72
0.95
3.00
11.95
8.0
7.0
6.0
5.0
4.0
3.0
2.0
1.0
ppm (t1)
10000
5000
0

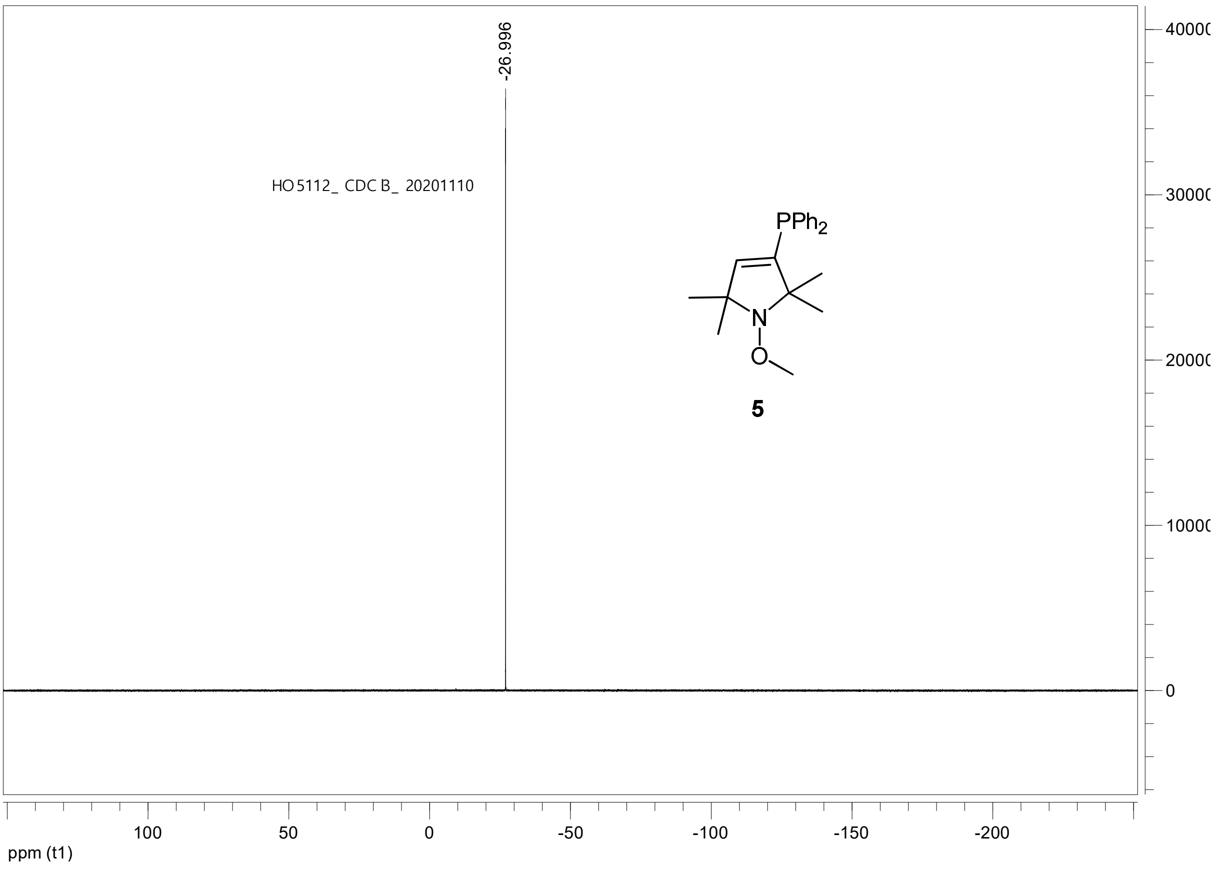

-26.996
HO5112_ CDC B_ 20201110
PPh2
N
O
5
100
50
0
-50
-100
-150
-200
ppm (t1)
40000
30000
20000
10000
0

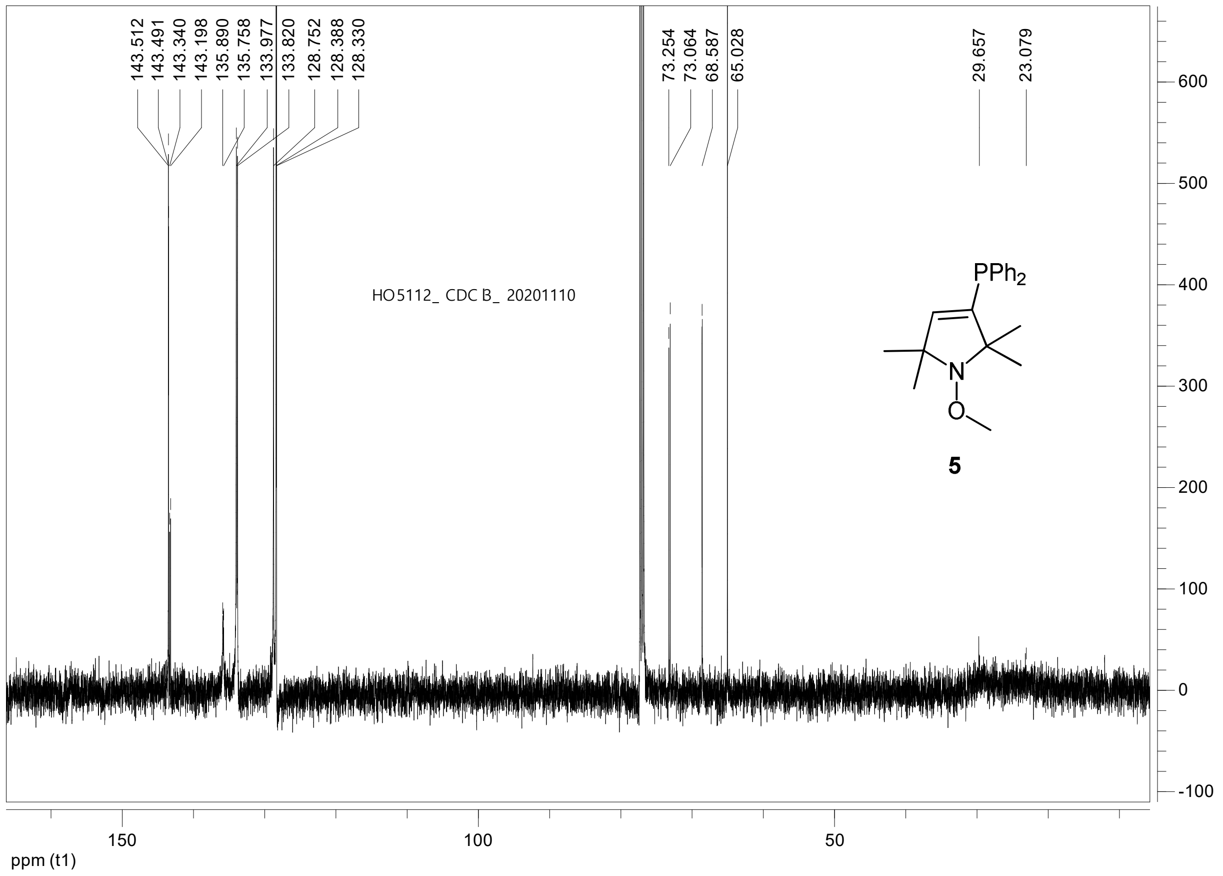

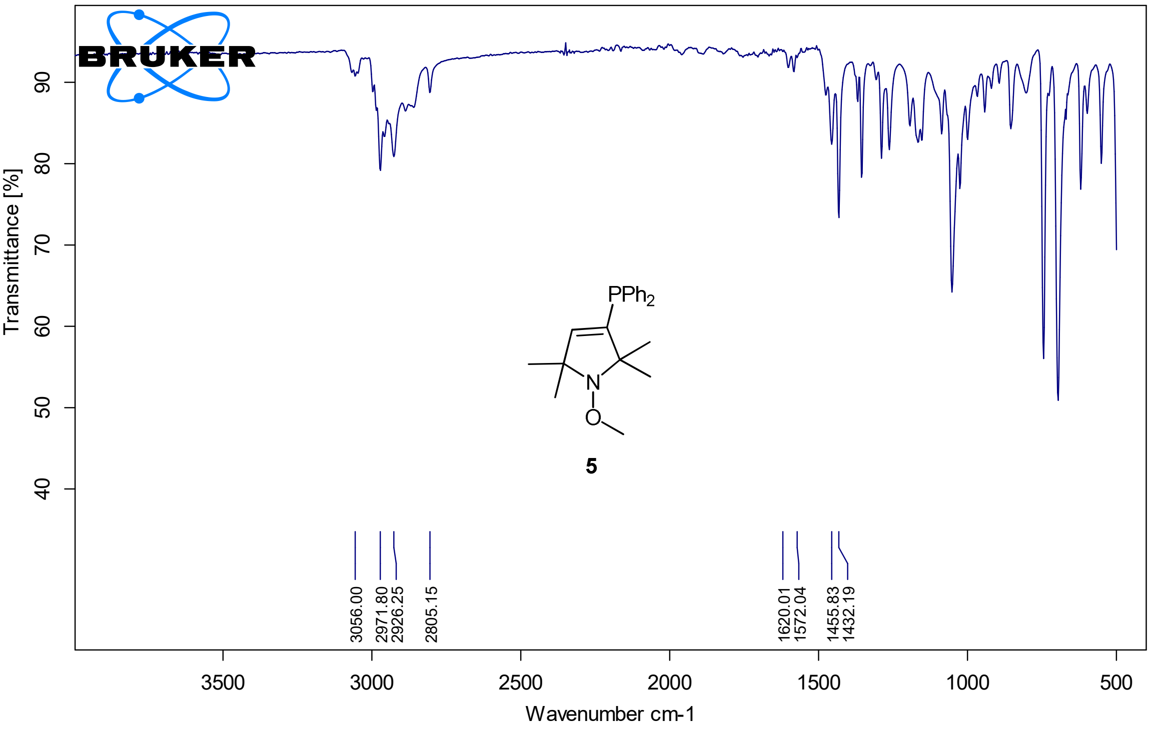

C:\OPUS_7.2.139.1294\Measure\HO-5012- OMe.0 HO-5012- OMe Instrument type and / or accessory	2021.03.29

Page 1/1

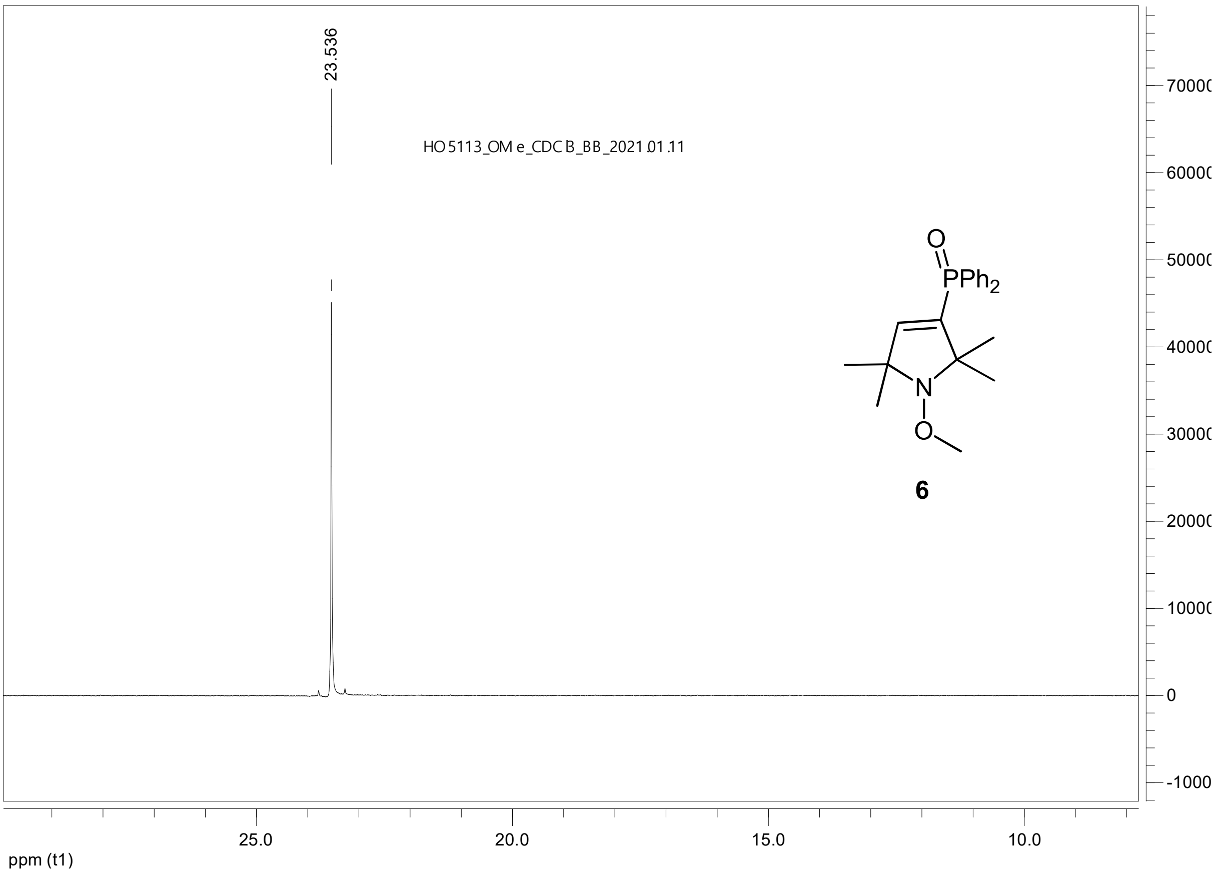
23.536
HO5113_OMe_CDCl3_BB_2021.01.11
PPh2
N
O
6
70000
60000
50000
40000
30000
20000
10000
0
-1000
25.0
20.0
15.0
10.0
ppm (t1)

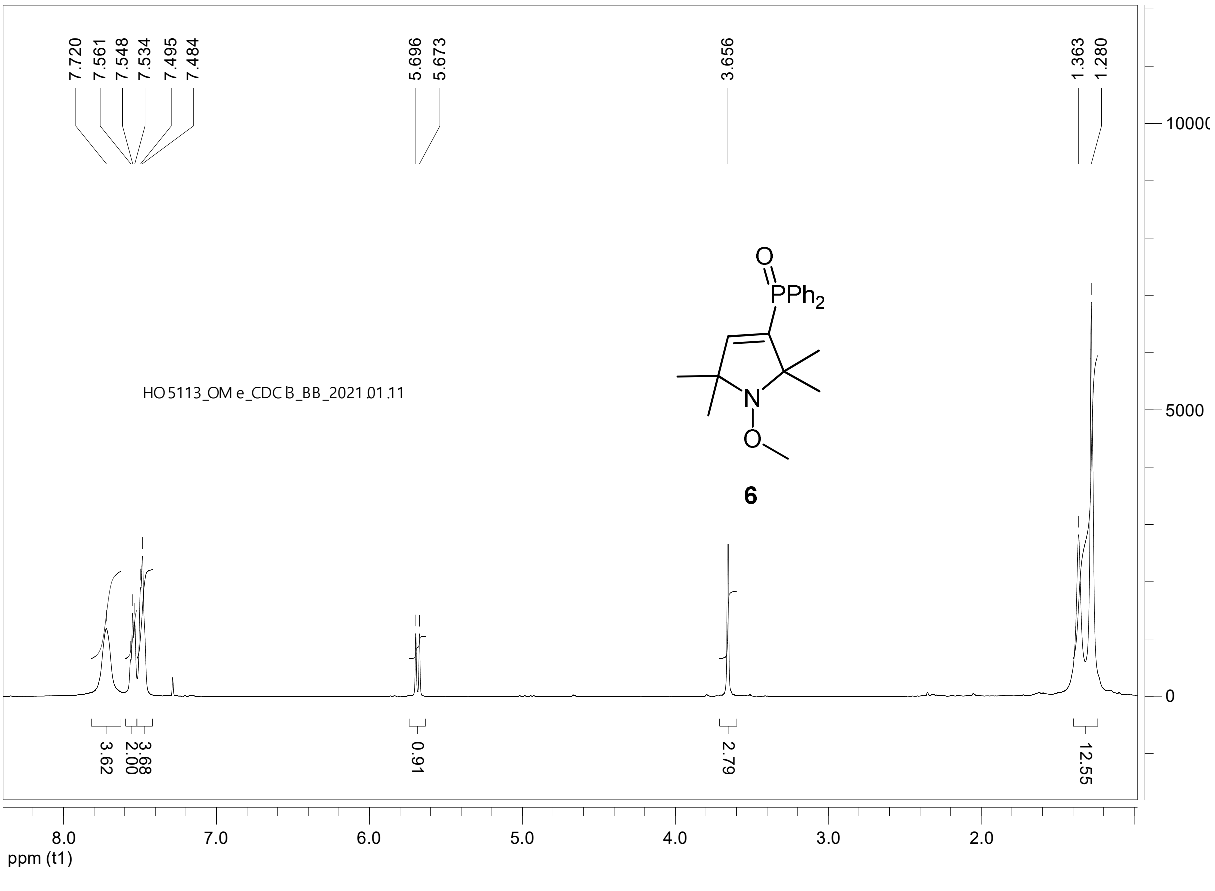
7.720
7.561
7.548
7.534
7.495
7.484
5.696
5.673
3.656
1.363
1.280
HO5113_OMe_CDCl3_BB_2021.01.11
PPh2
N
O
6
3.62
2.00
3.68
0.91
2.79
12.55
10000
5000
0
8.0
7.0
6.0
5.0
4.0
3.0
2.0
ppm (t1)

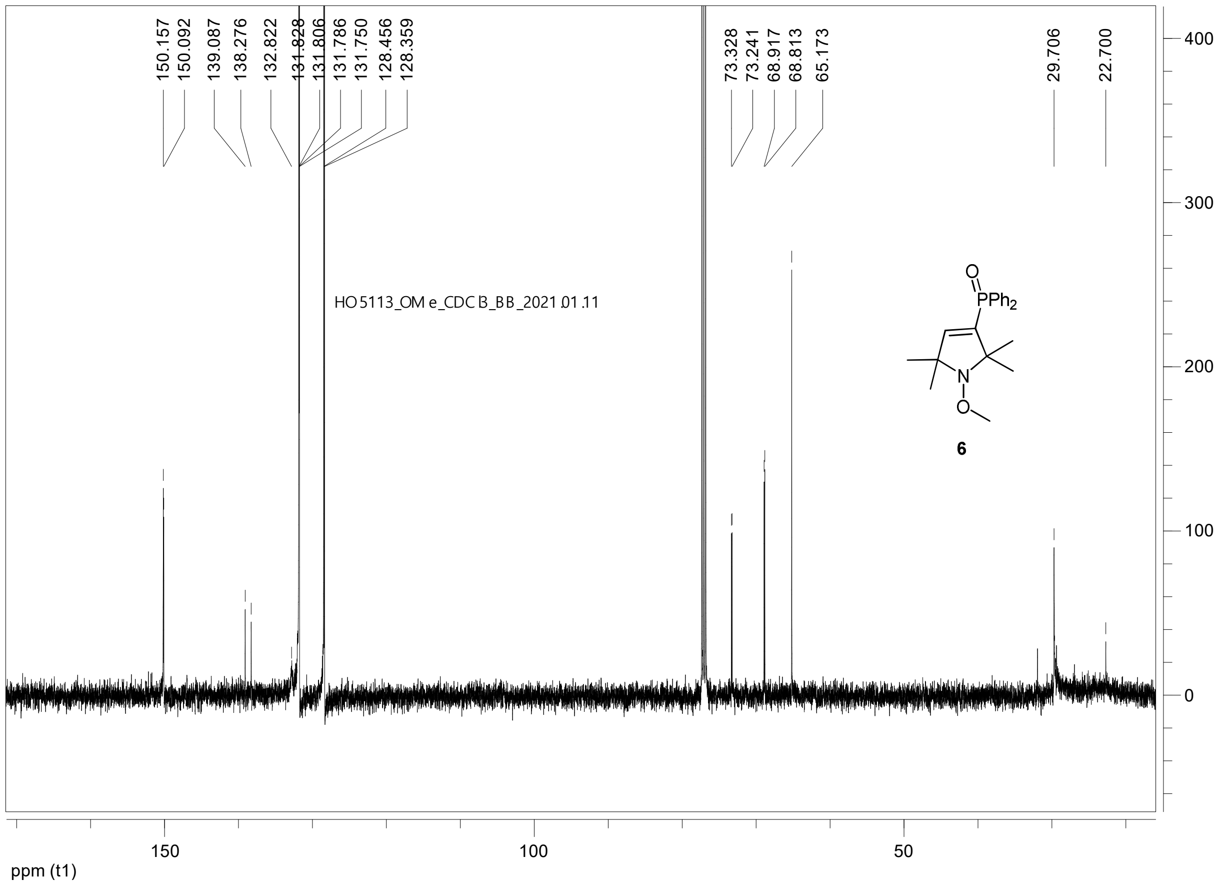

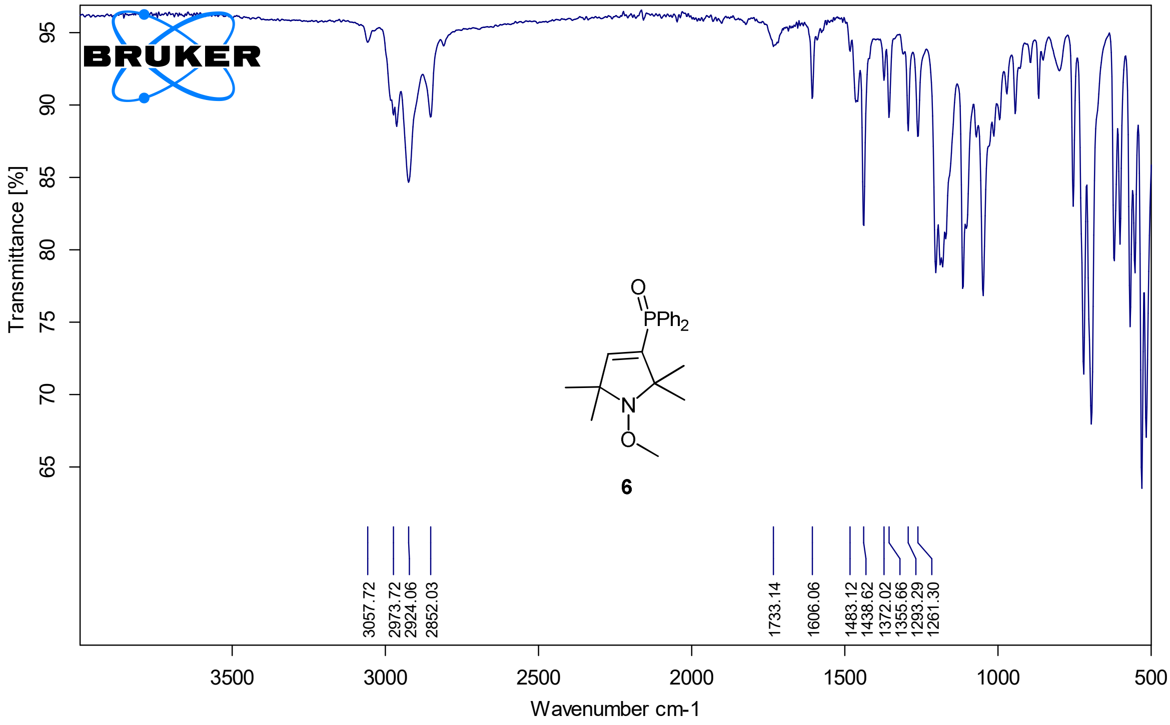

C:\OPUS_7.2.139.1294\Measure\HO-5113-OMe.0	HO-5113-OMe	Instrument type and / or accessory	2021.01.25

Page 1/1

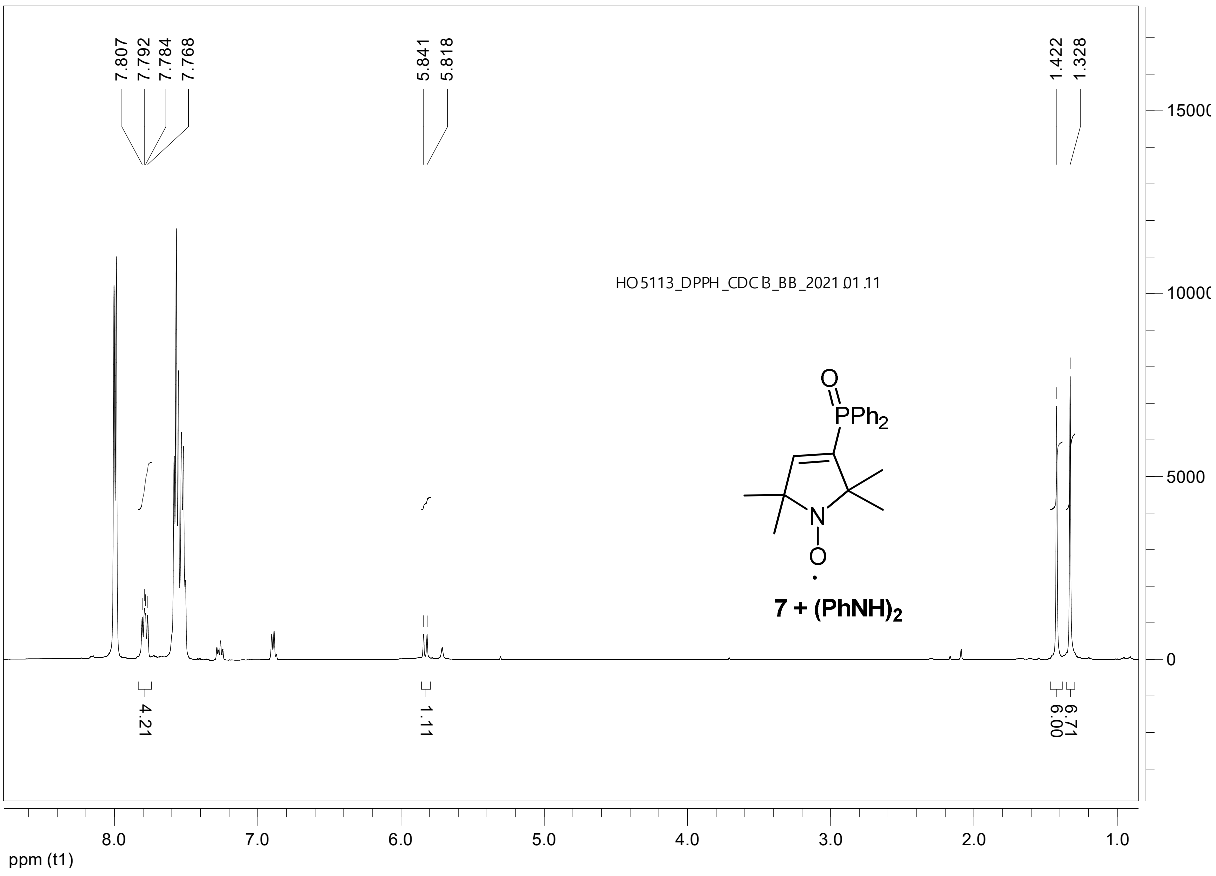
7.807
7.792
7.784
7.768
5.841
5.818
1.422
1.328
HO5113_DPPH_CDCl3_BB_2021.01.11
PPh2
7 + (PhNH)2
4.21
1.11
6.00
6.71
15000
10000
5000
0
8.0
7.0
6.0
5.0
4.0
3.0
2.0
1.0
ppm (t1)

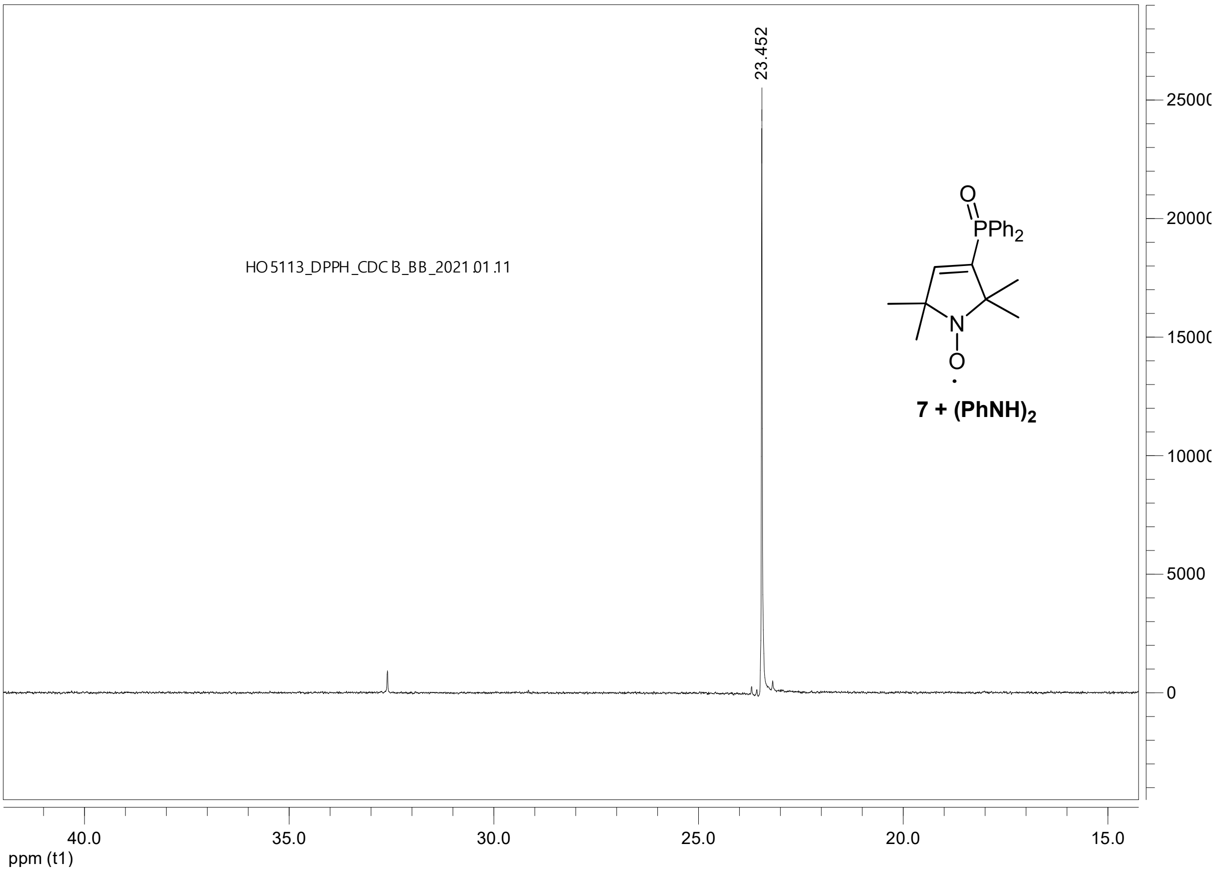
23.452
HO5113_DPPH_CDCl3_BB_2021.01.11
PPh2
7 + (PhNH)2
25000
20000
15000
10000
5000
0
40.0
35.0
30.0
25.0
20.0
15.0
ppm (t1)

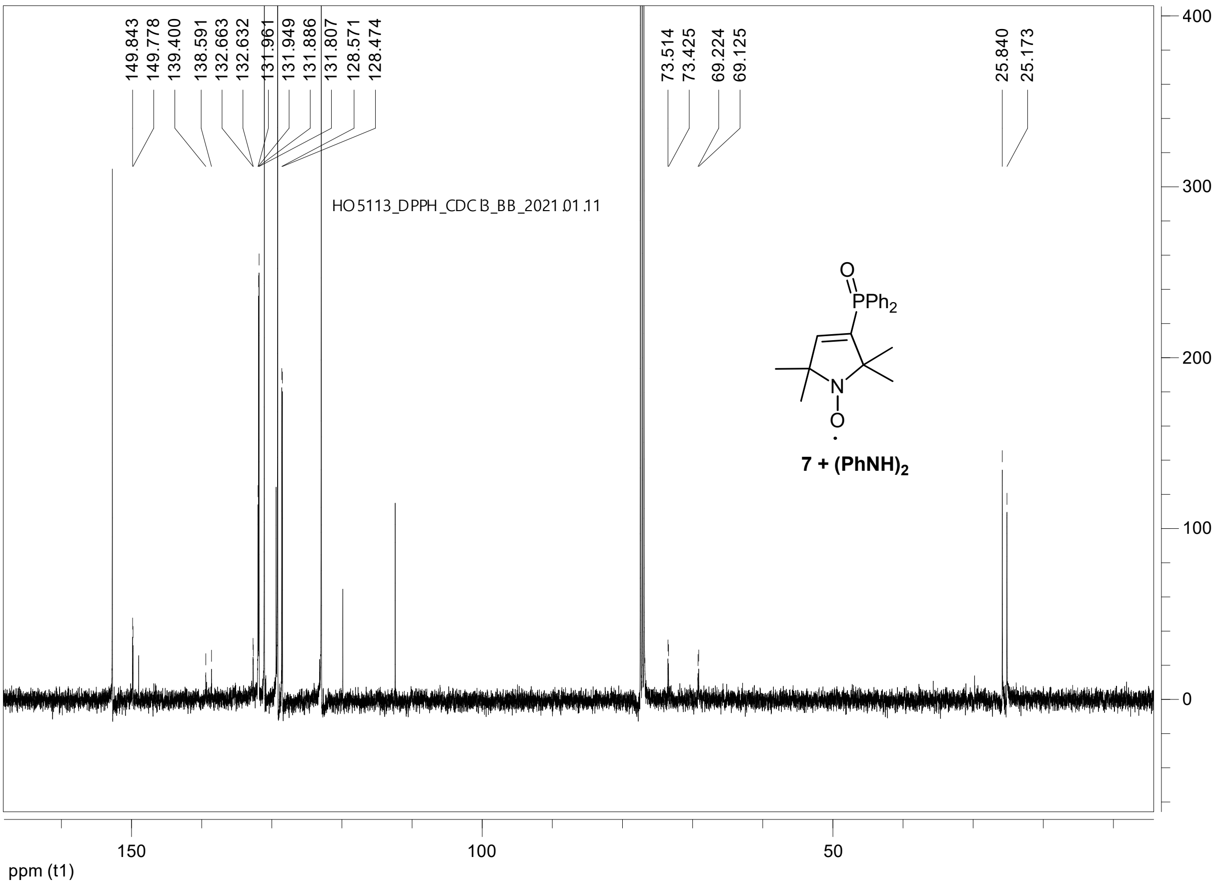

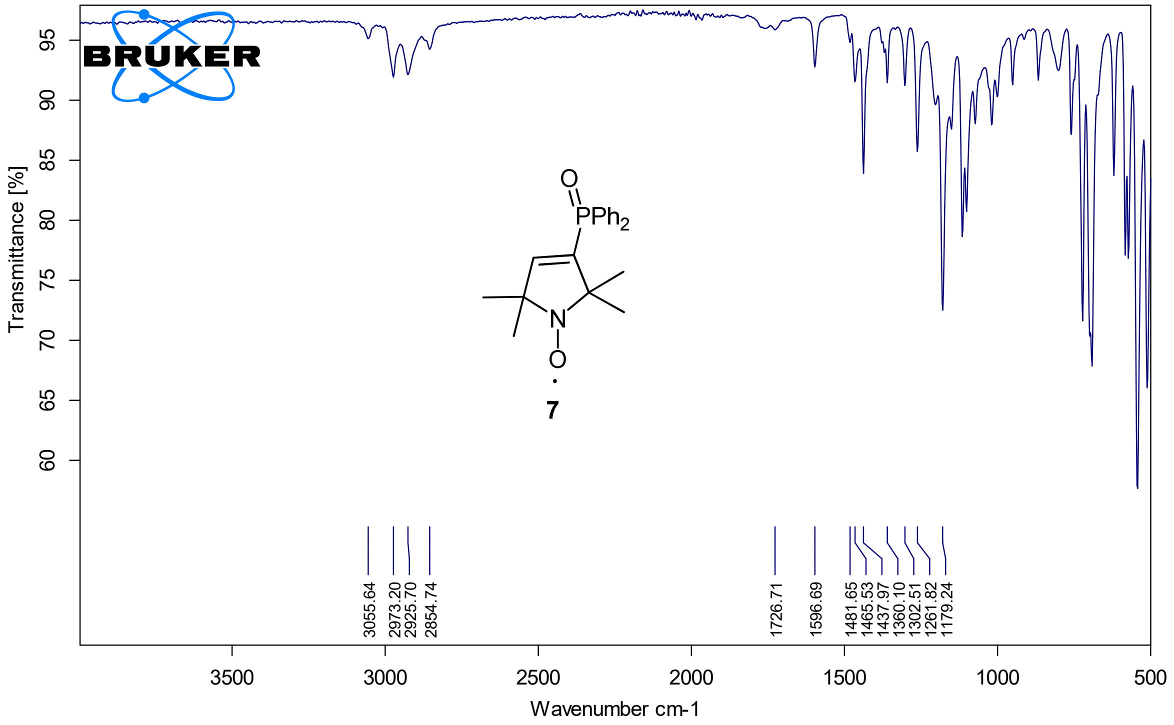

C:\OPUS_7.2.139.1294\Measure\HO-5113.0	HO-5113	Instrument type and / or accessory	2021.01.25

Page 1/1

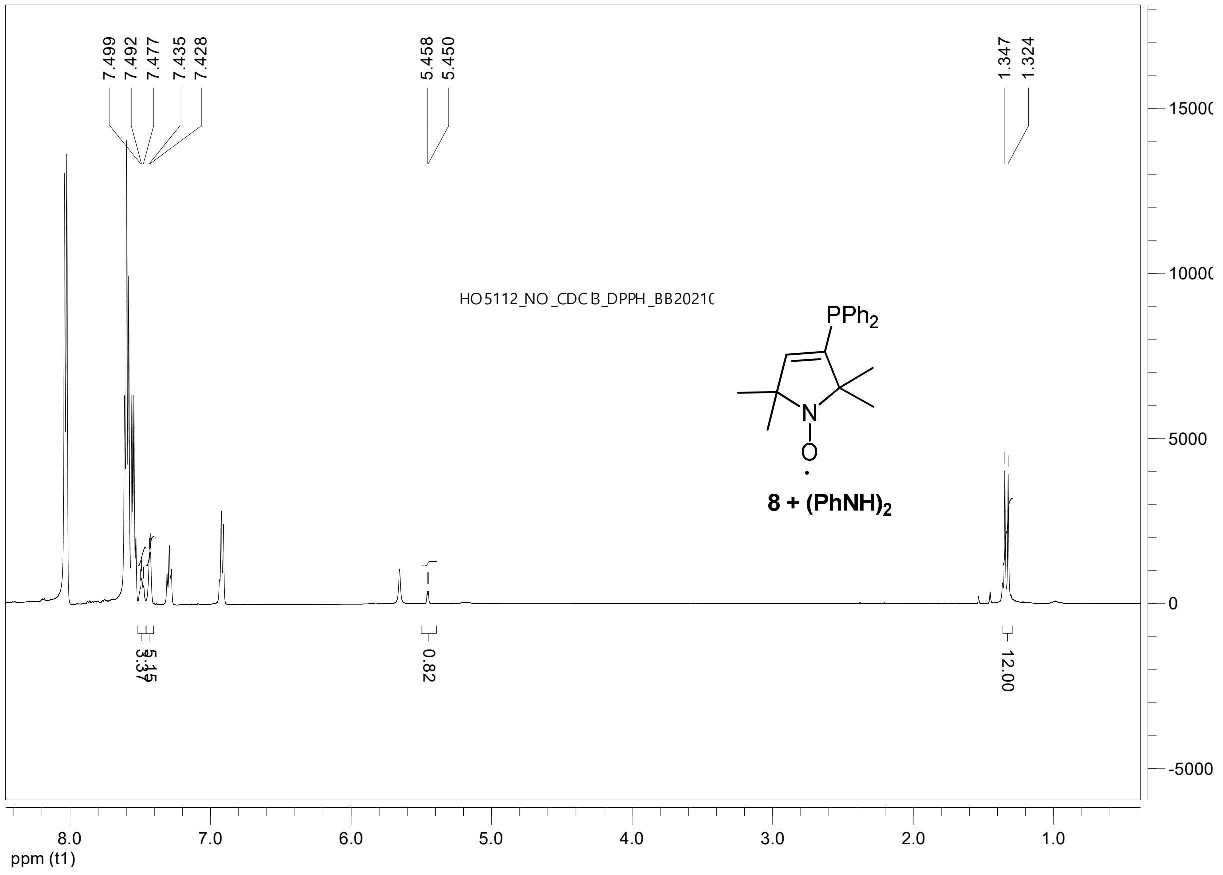
7.499
7.492
7.477
7.435
7.428
5.458
5.450
1.347
1.324
HO5112_NO_CDCB_DPPH_BB2021(
PPh2
N
O
8 + (PhNH)2
3.37
5.15
0.82
12.00
15000
10000
5000
0
-5000
8.0
7.0
6.0
5.0
4.0
3.0
2.0
1.0
ppm (t1)

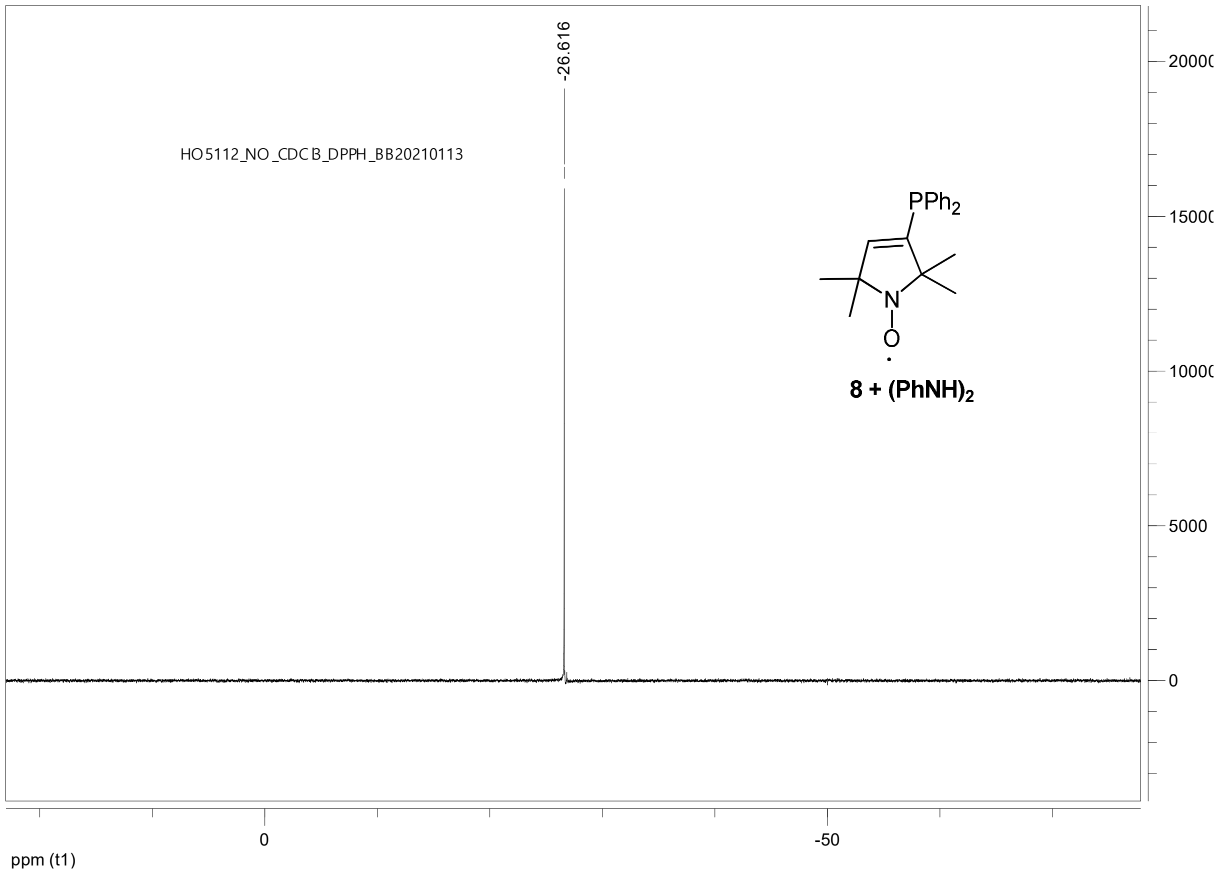
-26.616
HO5112_NO_CDCB_DPPH_BB20210113
PPh2
N
O
8 + (PhNH)2
20000
15000
10000
5000
0
0
-50
ppm (t1)

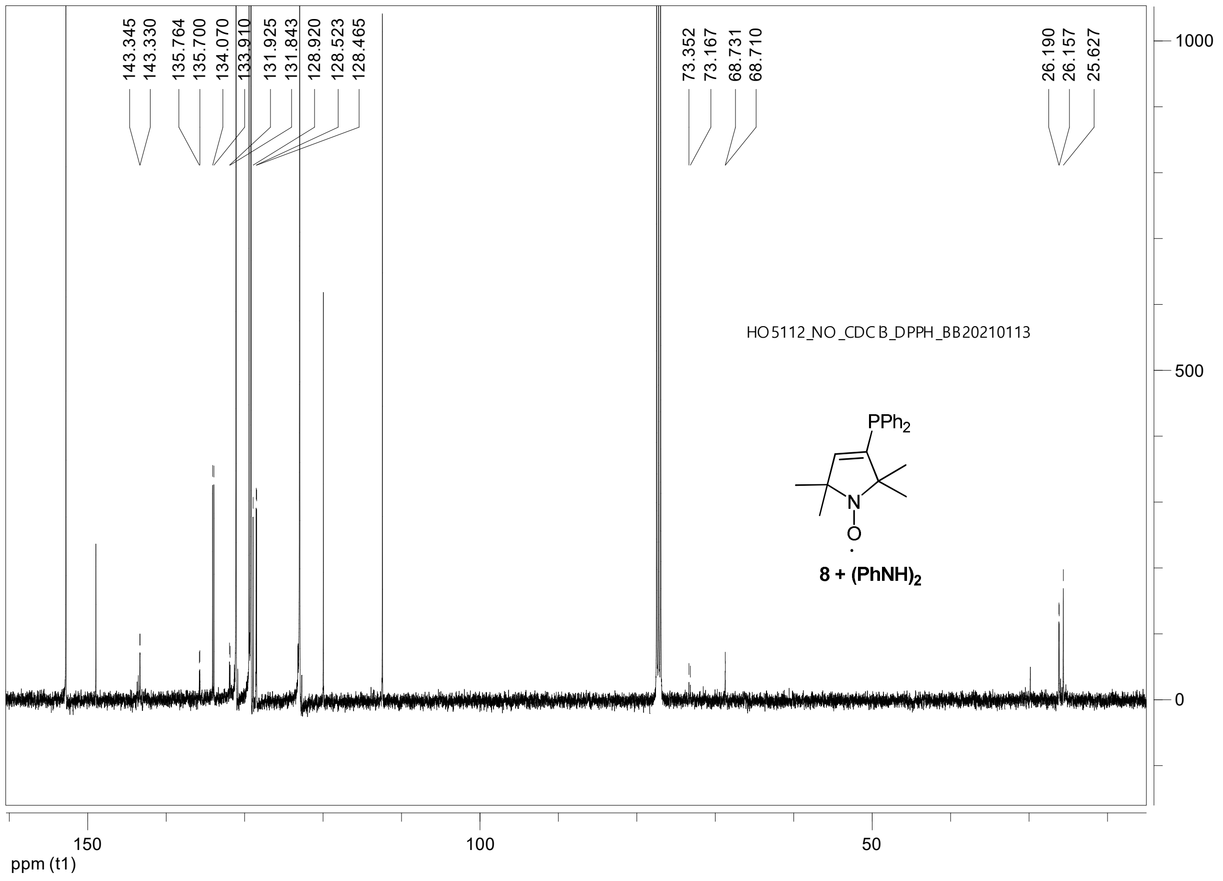

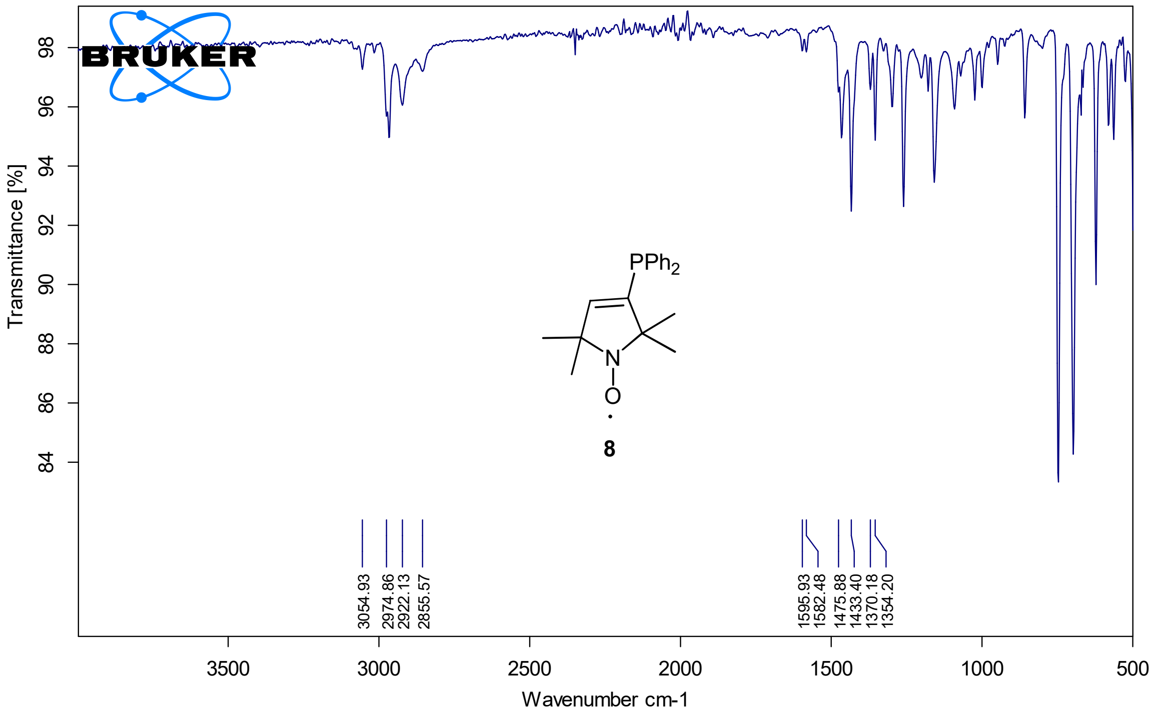

C:\OPUS_7.2.139.1294\Measure\HO-5112-radical.0 HO-5112-radical Instrument type and / or accessory	2021.01.25

Page 1/1

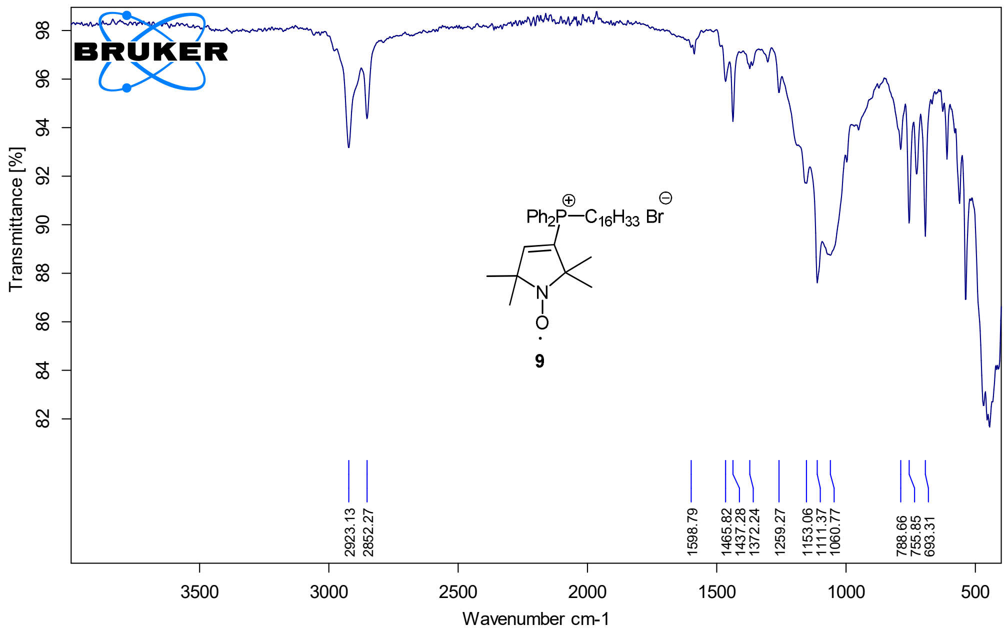

C:\OPUS_7.2.139.1294\Measure\ATR_Di\HO-5114-radical.1 HO-5114-radical Instrument type and / or accessory	2021.02.16

Page 1/1

EPR of compound **9**

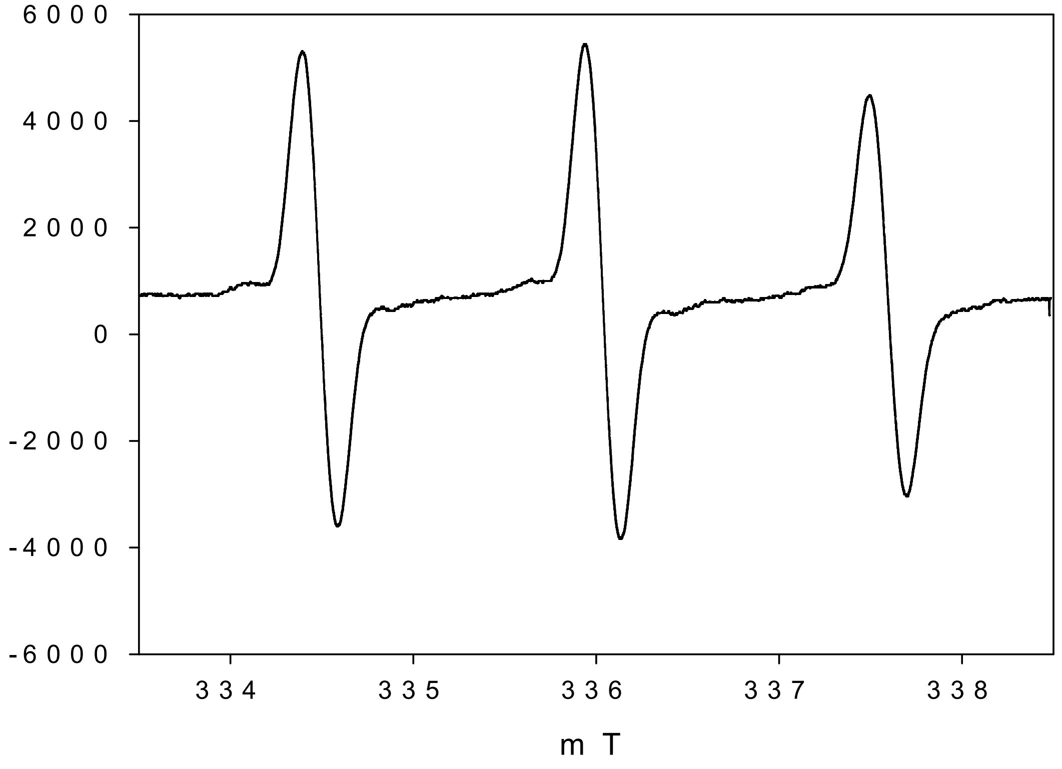

Thee EPR spectra of compound **9** (1×10^{-4} M) in water/dioxane 9:1.

EI-MS spectra of compound **1b:**

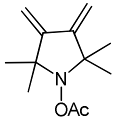

1b

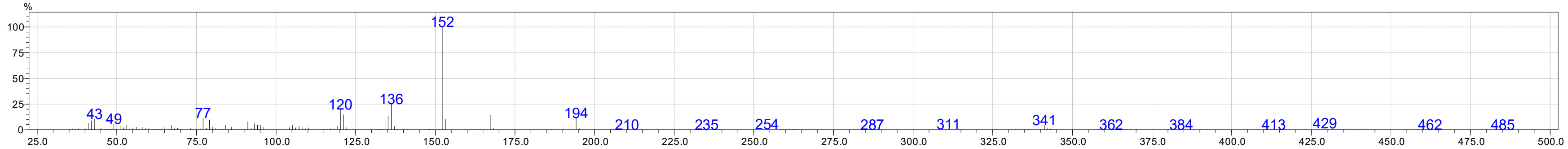

EI-MS spectra of compound **2b:**

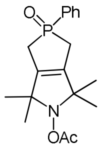

2b

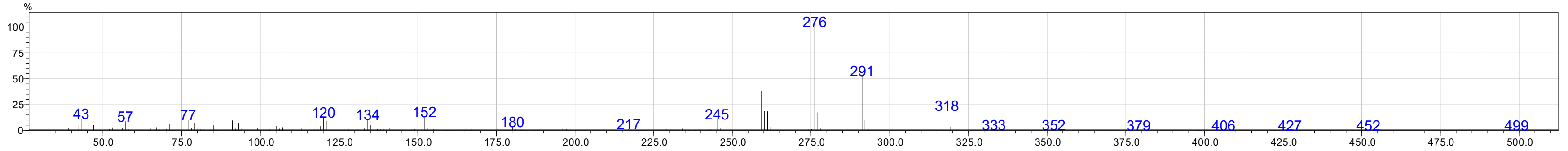

EI-MS spectra of compound **2a:**

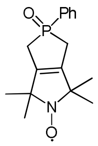

2a

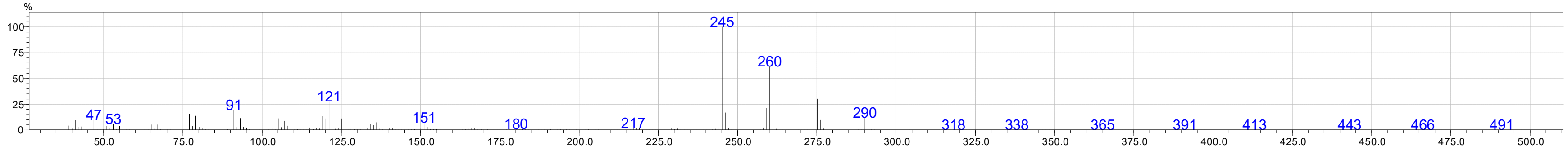

EI-MS spectra of compound **4:**

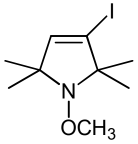

4

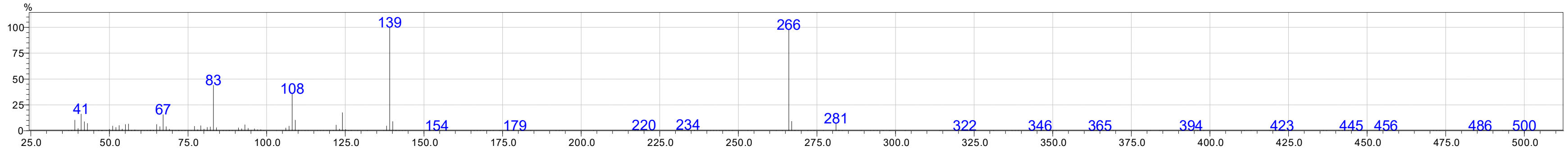

EI-MS spectra of compound **5**:

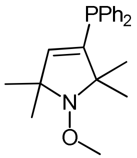

5

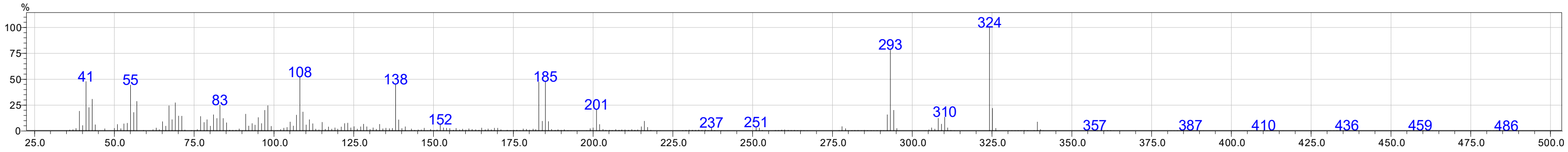

EI-MS spectra of compound **6**:

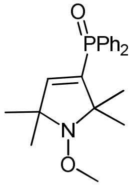

6

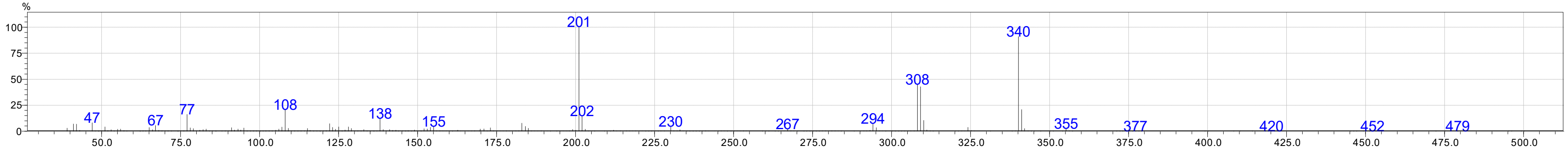

EI-MS spectra of compound **7**:

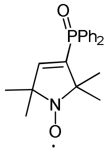

7

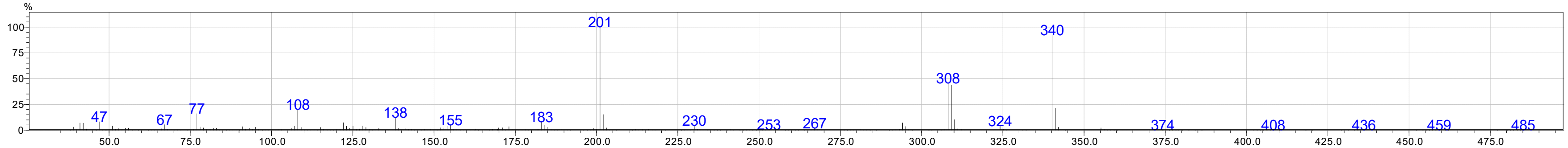

EI-MS spectra of compound **8:**

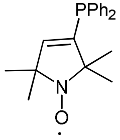

8

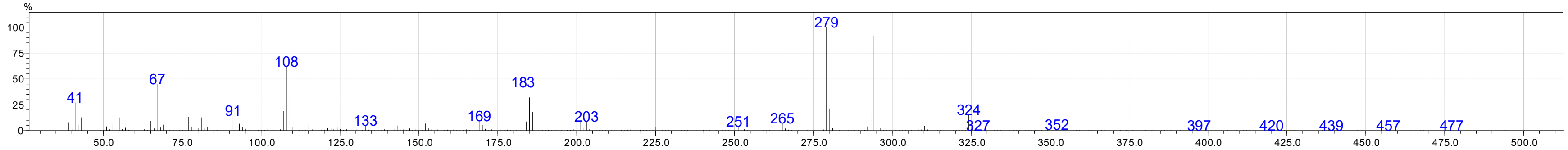

ESI-MS spectra of compound **9**:

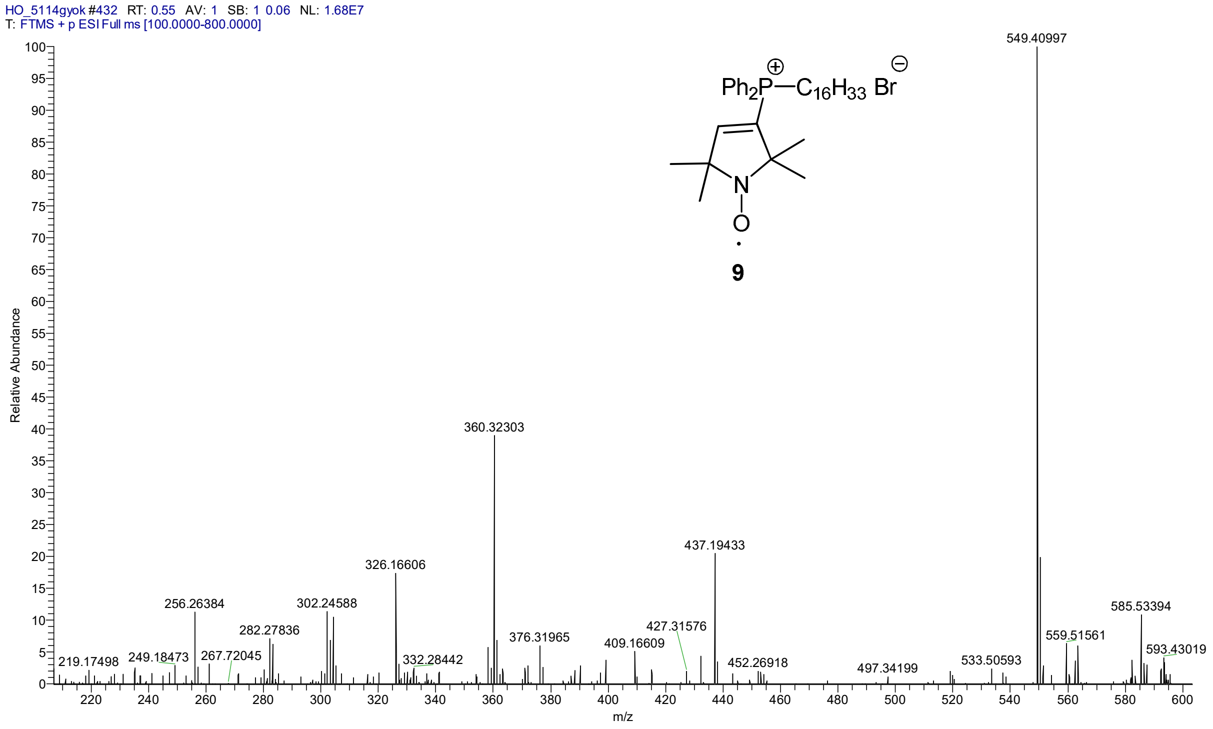

Table S1: Elememtal analysis of new compounds synthesized

Compound	Formula	Calculated %			Found %		
		C	H	N	C	H	N
**1b**	$C_{12}H_{19}NO_2$	68.87	9.15	6.69	68.75	8.95	6.59
**2a**	$C_{16}H_{21}NO_2P$	66.19	7.29	4.82	66.05	7.15	4.84
**2b**	$C_{18}H_{24}NO_3P$	64.85	7.26	4.20	64.65	7.15	4.17
**4**	$C_9H_{16}INO$	38.45	5.74	4.98	38.36	5.65	4.99
**5**	$C_{21}H_{26}NOP$	74.31	7.72	4.13	74.21	7.66	4.11
**6**	$C_{21}H_{26}NO_2P$	70.97	7.37	3.94	71.07	7.41	3.87
**7**	$C_{20}H_{23}NO_2P$	70.57	6.81	4.12	70.71	6.77	4.01
**8**	$C_{20}H_{23}NOP$	74.05	7.15	4.32	74.21	6.94	4.46
**9**	$C_{36}H_{56}BrNOP$	68.66	8.96	2.22	68.47	8.89	2.13

Figure S1: Preliminary biological data of compound 9 and cetyltriphenylphosphonium bromide (compound 10) compared to MITO-CP.

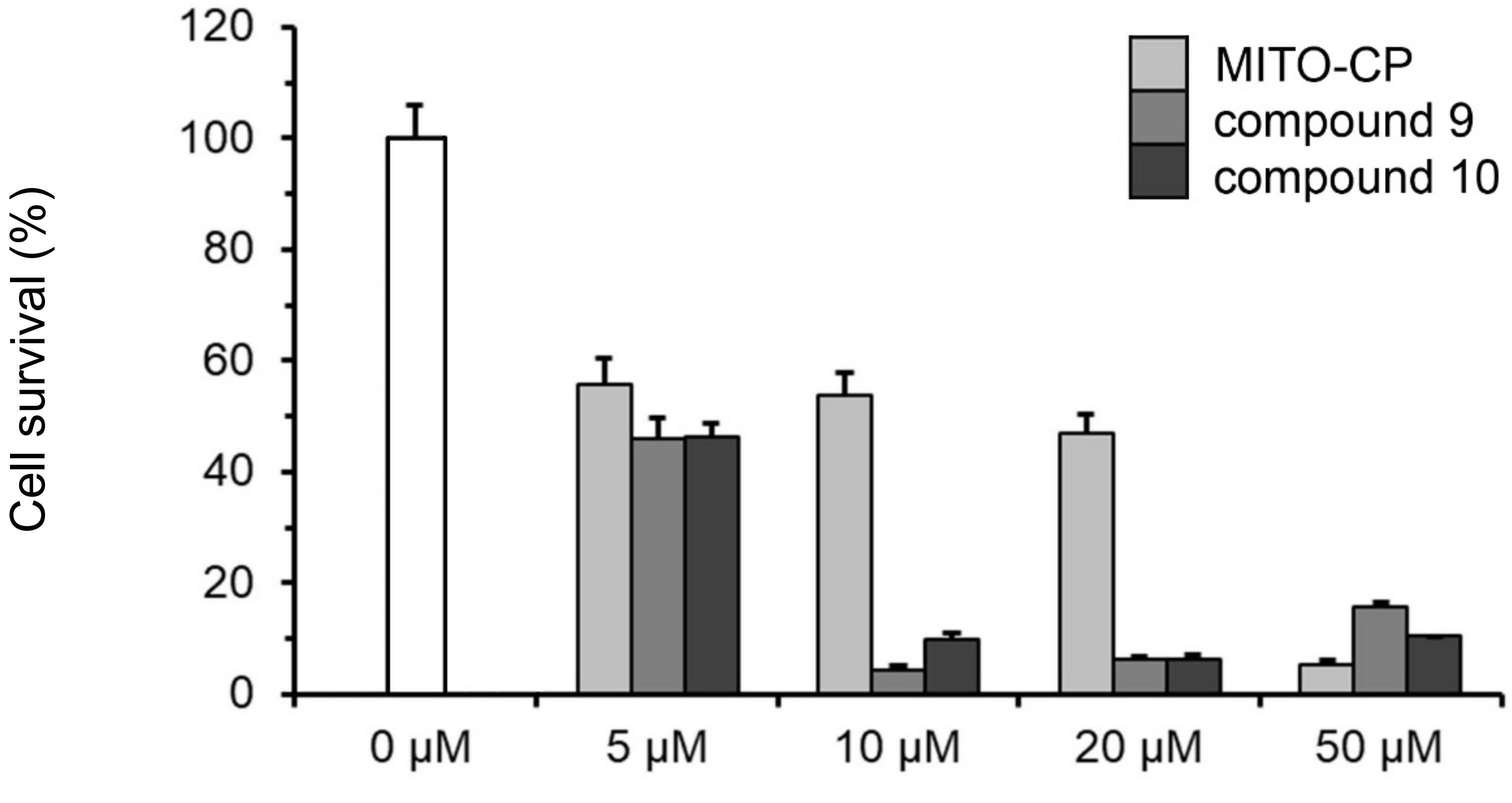

Supplementary Figure S1. Cytostatic effect of the substances on the MCF-7 human breast cancer line. The cells were treated with MITO-CP (light gray bars), compound **9** (gray bars) or compound **10** (dark gray bars) at the indicated concentrations for 24 h. Survival rate was is presented as the % of the untreated cells (empty bar) mean + standard error of the mean (SEM)of three independent experiments each running in at least four parallels (n=12).
